# Effect of different types of statins on kidney function decline and proteinuria: a network meta-analysis

**DOI:** 10.1038/s41598-019-53064-x

**Published:** 2019-11-12

**Authors:** K. Esmeijer, Olaf M. Dekkers, Johan W. de Fijter, Friedo W. Dekker, Ellen K. Hoogeveen

**Affiliations:** 1 Department of Nephrology, Leiden University Medical Center, Leiden, The Netherlands; 2 Department of Clinical Epidemiology, Leiden University Medical Center, Leiden, The Netherlands; 3 Department of Endocrinology, Leiden University Medical Center, Leiden, The Netherlands; 4 Department of Nephrology, Jeroen Bosch Hospital, Den Bosch, The Netherlands

**Keywords:** Risk factors, Kidney diseases

## Abstract

Previous studies showed that statins reduce the progression of kidney function decline and proteinuria, but whether specific types of statins are more beneficial than others remains unclear. We performed a network meta-analysis of randomized controlled trials (RCT) to investigate which statin most effectively reduces kidney function decline and proteinuria. We searched MEDLINE, Embase, Web of Science, and the Cochrane database until July 13, 2018, and included 43 RCTs (>110,000 patients). We performed a pairwise random-effects meta-analysis and a network meta-analysis according to a frequentist approach. We assessed network inconsistency, publication bias, and estimated for each statin the probability of being the best treatment. Considerable heterogeneity was present among the included studies. In pairwise meta-analyses, 1-year use of statins versus control reduced kidney function decline by 0.61 (95%-CI: 0.27; 0.95) mL/min/1.73 m^2^ and proteinuria with a standardized mean difference of −0.58 (95%-CI:−0.88; −0.29). The network meta-analysis for the separate endpoints showed broad confidence intervals due to the small number available RCTs for each individual comparison. In conclusion, 1-year statin use versus control attenuated the progression of kidney function decline and proteinuria. Due to the imprecision of individual comparisons, results were inconclusive as to which statin performs best with regard to renal outcome.

## Introduction

Chronic kidney disease (CKD) is an increasing global health burden owing to population ageing and unhealthier lifestyle^1^. Up to 11% of the European population aged 45 y or older has CKD stage 3, defined as an estimated glomerular filtration rate (eGFR) below 60 mL/min/1.73 m^2^ ^2^. CKD is an independent risk factor for cardiovascular morbidity and mortality^3^. Nowadays, the most important causes of CKD are cardiovascular disease, hypertension, diabetes, smoking, and hypercholesterolemia^4,5^. Generally, patients with symptomatic cardiovascular disease are prescribed cholesterol-lowering medication for secondary cardiovascular prevention. The latest KDIGO guideline on lipid management in CKD, recommends treatment with a statin in all non-dialysis dependent CKD patients ≥50 years with an eGFR below 60 mL/min/1.73 m^2^ or with at least 30 mg/g albuminuria, independent of serum cholesterol levels, which is also stated by the 2016 ESC/EAS guidelines^6,7^. Younger patients should use a statin in case of elevated cardiovascular risk, such as diabetes or coronary heart disease. Finally, statins should be continued, but not initiated, in patients on dialysis^6^. Multiple meta-analyses studied the effect of statins on renal outcomes. Recently, a meta-analysis by Su *et al*. concluded that statin users *vs* nonusers have a slower rate of kidney function decline and less proteinuria^8^.

Targeted prevention of kidney function decline is important to improve life expectancy and quality of life. However, it remains unclear whether specific types of statins are more beneficial than others regarding slowing down kidney function decline and lowering proteinuria. Various statins have different characteristics in terms of half-life, structure, lipophilicity, and potency^9^. We therefore performed a network meta-analysis of randomized controlled trials in adults that compare any statin with another statin or control treatment, to investigate which statin most effectively reduces kidney function decline or proteinuria. Network meta-analyses take into account both direct and indirect evidence of multiple comparisons in a treatment network, and provide information on which treatment performs best. These results may inform future guidelines about prevention of CKD and slowing down its progression.

## Methods

### Systematic literature review

We performed a systematic review of the literature, searching MEDLINE, Embase, Web of Science, and the Cochrane Library, on July 13^th^, 2018. Eligible studies were randomized controlled trials (RCT) in adults (patients ≥ 18 years) with a follow-up duration of at least one year, that included at least 10 patients per trial arm, and reported on changes in eGFR and/or proteinuria. The intervention of interest was statin therapy, the comparator either another statin, no intervention, cholesterol lowering diet, or placebo. In the entire manuscript, control treatment refers to any non-statin intervention. Combination therapy of statin with ezetimibe was also considered. A detailed outline of the search strategy is provided in the Supplemental Data, Appendix. Titles and abstracts were screened and relevant articles were read in full by two reviewers (KE and EH). Conference abstracts were excluded. No language restrictions were imposed. Post-hoc analyses of RCTs were only included when outcomes according to the original randomization group could be derived. In case of duplicate publications, we selected the publication that reported the data of interest most completely. References of included studies were additionally screened for relevant RCTs. We reported the results according to the Preferred Reporting Items for Systematic Reviews and Meta-analyses (PRISMA) guidelines for network meta-analyses^10^. The protocol for this meta-analysis was registered at PROSPERO: registration number CRD42018099613^11^.

### Outcome measures

The outcomes of interest were annual change of estimated glomerular filtration rate (eGFR) and proteinuria. Kidney function estimates calculated by the Cockroft-Gault formula, the Modification of Diet in Renal Disease (MDRD) formula, or Chronic Kidney Disease Epidemiology Collaboration (CKD-EPI) equation were pooled. If change of kidney function or proteinuria was not reported, it was calculated by subtracting the baseline value from follow-up. The standard deviation (SD) of change was calculated using the SDs of eGFR or proteinuria at baseline and follow-up, according to the following formula^12^:

$${{\rm{SD}}}_{change}=\sqrt{{{{\rm{SD}}}_{0}}^{2}+{{{\rm{SD}}}_{1}}^{2}-(2\ast Corr\ast {{\rm{SD}}}_{0}\ast {{\rm{SD}}}_{1})}$$

where SD_0_ and SD_1_ represent the SD of baseline and follow-up, respectively, and *Corr* represents a correlation coefficient, which describes the similarity between baseline and follow-up measurements. The correlation coefficient was derived from studies that reported both baseline and follow-up eGFR or proteinuria with an SD, and change in eGFR or proteinuria with SD, according to the following formula^12^:

$$Corr=\frac{{{{\rm{SD}}}_{0}}^{2}+{{{\rm{SD}}}_{1}}^{2}+{{{\rm{SD}}}_{change}}^{2}}{2\ast {{\rm{SD}}}_{0}\ast {{\rm{SD}}}_{1}}$$

Based on data from three intervention studies investigating the effect of statins on kidney function, and data from the Alpha Omega Trial, we assumed a correlation coefficient between baseline and follow-up eGFR of 0.8^13–16^. In the main analysis we compared change of eGFR or proteinuria after 12 months for statin users *vs* control treatment. If no data were reported on change in eGFR or proteinuria after one year, we used the available data to calculate an annual change assuming a linear decline in line with the results of a recent study^17^.

### Data extraction and quality assessment

Data extraction was performed by two independent reviewers (KE and EH) who used a standard form. Discrepancies were resolved by discussion or by consulting a third reviewer (OD). We extracted the following data: study name, study year, trial acronym, duration, population type, treatment arms, sample size, mean age, sex (% males), diabetes (%), hypertension (%), mean systolic and diastolic blood pressure, use of renin-angiotensin system (RAS) blocking drugs (%), low-density lipoprotein (LDL) level at baseline and follow-up, baseline and follow-up eGFR, change in eGFR, baseline and follow-up proteinuria, and change in proteinuria. When the outcome of interest was not reported in a table or text, we extracted the exact numbers from figures.

The Cochrane Collaboration Risk of Bias tool was used to assess potential sources of bias: selection, performance, detection, attrition and reporting bias^18^. We scored per included RCT each type of bias as follows: low, high, or unclear risk of bias. Risk of bias was scored high in case of broken randomization, absent blinding of participants, absence of allocation concealment, and in case of large number of missing outcome data, or exclusion of patients. Since the outcome of interest was based on laboratory measurements, we considered for all RCTs, including the open-label RCTs, the risk of bias “low” with regard to blinding of outcome assessment.

### Statistical analysis

First, we performed a pairwise random-effects meta-analysis for the effect of statin *vs* control on eGFR and proteinuria decline. For eGFR decline we used the weighted mean difference (WMD) as measure for the pooled estimates. For proteinuria we estimated standardized mean differences (SMD) to account for different methods to express proteinuria: urinary albumin to creatinine ratio, urinary protein excretion, urinary albumin excretion, or log-transformed protein excretion. Statistical heterogeneity was assessed by the I^2^-statistic, which quantifies the variation across studies due to heterogeneity rather than chance^19^. We used meta-regression to evaluate whether heterogeneity could be explained by age, sex, diabetes, blood pressure, baseline LDL, change in LDL, or risk of bias. Finally, we assessed the presence of publication bias visually with a funnel plot and formally by the Egger’s test^20,21^. This rank-based method estimates the number and outcomes of missing unpublished studies, and adjusts the estimate after incorporating these theoretical studies.

Second, we performed a random-effects network meta-analysis, following a frequentist approach. In case multiple dosages were reported, we analyzed high and low statin dosages as separate treatments. We took as outcome the WMD of annual kidney function decline and change of proteinuria expressed as SMD. We checked for transitivity and consistency. Transitivity was judged clinically; consistency was judged formally^22^. We tested for possible inconsistency globally using a χ^2^-test, and locally by calculating inconsistency factors for each comparison in closed loops. In case of minor inconsistencies, possible reasons for inconsistency were considered. Furthermore, we estimated for each statin, compared to control, the treatment effect with 95%-confidence intervals and prediction intervals. The prediction interval represents the expected range of true effects in similar (future) studies, and will be broader than the confidence interval in case of high heterogeneity^23^. Finally, for each statin, with or without ezetimibe, we calculated the surface under the cumulative ranking (SUCRA) line. We used the SUCRA to provide a hierarchic overview of treatments, and to give an impression of the most efficacious treatments^24^. The SUCRA takes into account for every treatment the cumulative probabilities of all possible rankings. If a treatment always ranks first, the SUCRA is 100% (or 1), and 0% (or 0) if it always ranks last^25^.

We repeated the analyses excluding RCTs with a total sample size <100 patients or stratified by open-label (yes/no) or post-hoc (yes/no) status. Subgroup analyses were not considered if too few RCTs remained to form a network. All statistical analyses were performed using STATA Statistical Software version 14 (Statacorp, Texas, USA), and the *StataNMA* package^26^.

## Results

### Characteristics of included studies

**Figure 1:**
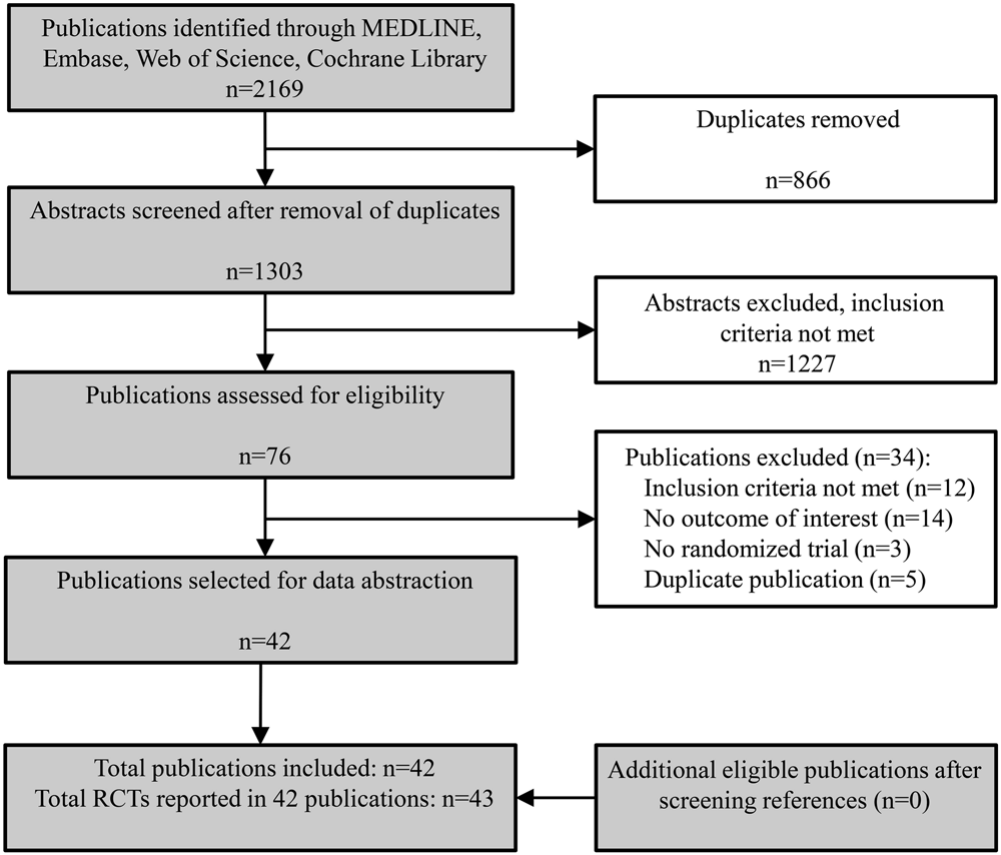
Flow chart of literature search and included full text publications. All included publications were included in quantitative analyses, depending on the reported endpoint(s).

After removing duplicate RCTs, 1303 titles and abstracts were screened for eligibility; 76 full publications were assessed. Finally, 43 RCTs comprising over 110,000 patients reported in 42 publications were included (Fig. 1). Of these 42 publications, 40 were in English, one was Russian^27^, and one Japanese^28^. In total, 40 RCTs reported about the effect of statins on change of eGFR^13–15,27,29–63^, of which 30 compared a statin to control, and 10 compared two or more statins with each other. The effect of statins on proteinuria was reported in 25 RCTs^13,14,28,29,32–34,36,39,45,46,48–54,57,60,62–65^, of which 19 compared a statin to control intervention, and six compared two or more statins. Characteristics of included RCTs are shown in Table 1. The included RCTs investigated seven different statins with varying dosages, and in three RCTs a statin was combined with ezetimibe^40,46,48^. Of all included RCTs, 11 comprised coronary heart disease patients, 11 comprised CKD patients, and 11 comprised diabetes mellitus type 2 patients. The mean age of the enrolled patients in most RCTs was over 50 years and about 66% were men. The unweighted mean (range) of baseline LDL-cholesterol from all individual RCTs was 3.7 (2.2–7.8) mmol/L, and statin compared to control treatment led to a mean (SD) 27% (9%) reduction of the serum LDL level. The majority of RCTs had a low risk of bias (Supplementary Fig. S1). However, about a 44% of all RCTs was open-label and about 25% were post-hoc analyses.

**Table 1:** characteristics of included studies.

Author, year Study name	Population	Intervention	Mean baseline characteristics per RCT								Outcome [annual change (SD)] per intervention	
			Sample size (n)	Follow-up (y)	Age (y)	Male sex (%)	Diabetes (%)	Blood pressure (mmHg)	eGFR (mL/min/1.73m^2^)	LDL (mmol/L)	eGFR	Proteinuria
**(measure)**												
Abe, 2015	CKD	Rosuvastatin 2.5 mg Pitavastatin mean 1.4 mg	134	1	70	58	44	.	58	3.6	2.80 (12.1) 0.90 (13.8)^a^	-392 (802) -250 (707)^a^ (UACR)
Amarenco, 2014 SPARCL	Stroke, TIA	Atorvastatin 80 mg Placebo	4719	5	63	60	17	139/82	66	3.4	0.96 (13.1) -0.50 (13.1)^b^	.
Athyros, 2004 GREACE	CHD	Atorvastatin mean 24 mg Control	1600	4	58	78	20	123/75	77	.	2.00 (2.0) -0.75 (1.8)^a^	.
Atthobari, 2006 PREVEND-IT	General population	Pravastatin 40 mg Placebo	788	4	52	66	3	131/76	76	4.1	0.15 (3.7) -0.25 (1.9)^a^	-0.02 (0.07) -0.03 (0.08)^ac^ (UAE)
Bianchi, 2003	CKD	Atorvastatin 40 mg Placebo	56	1	56	47	0	133/85	50	5.1	-1.00 (5.9) -5.80 (6.0)^a^	-1.0 (0.47) 0.3 (0.47)^a^ (UPE)
Castelao, 1993	Transplant	Lovastatin 20 mg Simvastatin 10 mg	51	1	44	69	.	.	52	4.9	-1.00 (16.6) -4.60 (15.3)	0.38 (1.9) 0.31 (1.1) (UPE)
Colhoun, 2009 CARDS	DM2	Atorvastatin 20 mg Placebo	2838	4	62	68	100	144/83	64	3.0	0.48 (2.7) 0.30 (2.6)	.
Dalla Nora, 2003	DM2	Atorvastatin 10 mg Placebo	25	1	65	60	100	.	.	3.5	.	2.0 (1.9) 6.0 (1.9)^d^ (AER)
Deedwania, 2015 SAGE	CAD	Atorvastatin 80 mg Pravastatin 40 mg	868	1	72	69	23	.	62	3.8	2.38 (10.4) 0.18 (10.3)^b^	.
Fassett, 2010	ADPKD	Pravastatin 20 mg Control	60	2	51	39	.	133/86	55	3.3	-0.31 (10.4) -1.34 (12.2)	-0.04 (0.20) 0.01 (0.09) (UPE)
Fassett, 2010 LORD	CKD	Atorvastatin 10 mg Placebo	132	3	60	65	8	143/81	31	3.4	-1.04 (3.84) -1.47 (3.74)	-0.39 (0.71) -0.14 (0.85) (UPE)
Fellstrom, 2004 ALERT	Transplant	Fluvastatin 40 mg Placebo	439	5	50	66	19	144/86	52	4.1	-0.93 (8.9) -1.87 (8.3)^a^	.
Fried, 2001	DM1	Simvastatin 10 mg Placebo	39	1.5	32	56	100	.	.	3.3	.	0.09 (0.44) 0.14 (0.66)^d^ (AER)
Gheith, 2002	Nephrotic syndrome	Fluvastatin 20 mg Control	43	1	23	42	.	.	107	7.8	-4.80 (28.8) -35.4 (29.4)^a^	-6.0 (2.3) -2.0 (2.4)^de^ (UPE)
Haynes, 2014 SHARP	CKD	Simvastatin 20 mg/eze Placebo	5037	4	63	62	23	139/80	27	2.9	-1.66 (3.5) -1.83 (3.5)	.
Holme, 2010 IDEAL	MI	Atorvastatin 80 mg Simvastatin 20 mg	8888	4.8	62	81	12	137.80	68	3.1	0.01 (2.7) 0.34 (2.7)	.
HPS, 2003 HPS	DM	Simvastatin 40 mg Placebo	20536	4.8	64	76	29	144/81	.	3.3	-1.23 (1.86) -1.40 (1.83)	.
Huskey, 2009 4S	CHD	Simvastatin 20 mg Placebo	3842	5.5	58	80	4	139/83	76	4.9	-0.34 (7.4) -0.41 (7.4)^f^	.
Kendrick, 2010 AFCAPS/Tex	Primary prevention	Lovastatin 20 mg Placebo	4994	5.3	58	85	2	138/78	87	3.8	-1.30 (3.5) -1.40 (3.5)	.
Kimura, 2017 ASUCA	CKD	Atorvastatin 5-20 mg Control	334	2	63	64	34	133/77	55	3.7	-1.15 (4.4) -1.30 (4.4)	0.3 (1.3) -0.2 (1.3) log(UAE)
Kimura, 2012	DM2	Pitavastatin 2 mg Pravastatin 10 mg	83	1	65	57	100	132/76	74	3.4	-2.0 (9.0) -0.5 (9.5)^b^	-50 (150) 25 (175)^b^ (UACR)
Kinouchi, 2013	Dyslipidemia	Fluvastatin 20 mg Fluvastatin 20 mg/eze	54	1	54	67	6	140/90	71	4.1	-4.10 (7.7) 4.10 (6.4)	22.5 (72.4) -44.7 (74.5)^d^ (UAE)
Koren, 2009 ALLIANCE	CHD	Atorvastatin mean 41 mg Control	2442	4.5	61	82	22	134/79	73	3.8	0.18 (6.4) -0.30 (7.2)	.
Kouvelos, 2015	Vascular surgery	Rosuvastatin 10 mg Rosuvastatin 10 mg/eze	262	1	71	90	30	.	65	3.8	-7.60 (10.1) -6.80 (10.7)^a^	0.9 (2.0) 0.5 (1.9)^ad^ (UPE)
Lam, 1995	NID-DM	Lovastatin 20-40 mg Placebo	34	2	56	56	100	.	84	4.2	-1.10 (5.7) -1.30 (3.6)^ab^	0 (0.1) 0.25 (0.2)^ab^ (UPE)
Lee, 2005	Controlled HT	Pravastatin 10 mg Placebo	61	1	49	68	0	121/73	87	3.2	13.0 (13.3) 4.0 (12.4)^a^	-673 (448) -7 (327)^ab^ (UPE)
Lemos, 2013	CKD	Rosuvastatin 10 mg Control	77	2	58	61	21	.	40	3.1	-1.15 (6.0) -2.50 (5.1)a	0.08 (0.18) 0.23 (0.26)^ad^ (UPE)
Mori, 1992	NID-DM	Pravastatin 10 mg Control	33	1	63	36	100	134/80	.	2.9	.	-50.5 (54.7) -5.4 (71.8)^a^ (UACR)
Mou, 2016	Chronic glom. nephritis	Pravastatin 20 mg Control	48	1.8	51	.	8	133/75	75	3.5	-1.08 (12.7) -4.33 (10.6)^ab^	-0.33 (0.9) -0.27 (0.9)^ab^ (UPE)
Nanayakkara, 2007 ATIC	CKD	Pravastatin 40 mg * Placebo	87	2	53	57	0	135/79	34	3.6	0 (4.3) 0.15 (4.3)^ab^	-0.1 (0.8) 0.2 (0.8)^a^ log(UAE)
Ohsawa, 2015	CKD	Pitavastatin 1-4 mg Control	28	1	62	71	33	130/78	49	3.6	-3.50 (3.21) -4.20 (2.96)^a^	-244 (574) -338 (1141)^a^ (UACR)
Rahman, 2008 ALLHAT	HT, HCh	Pravastatin 40 mg Control	10355	6	67	51	35	143/83	78	3.8	-1.45 (5.9) -1.65 (5.9)^a^	.
Rutter, 2011 PANDA	DM2	Atorvastatin 80 mg Atorvastatin 10 mg	119	2.5	64	83	100	.	67	3.1	1.0 (13.8) -3.0 (11.8)^ab^	.
Sawara, 2008	CKD	Rosuvastatin 2.5 mg Control	38	1	65	0	.	127/78	53	3.3	2.60 (12.3) -2.20 (10.6)^a^	-0.04 (0.19) 0.05 (0.24)^a^ (UPE)
Scanferla, 1991	CKD	Sim/pravastatin 10 mg Control	24	1	54	58	.	172/106	40	.	-1.80 (4.2) -3.10 (4.2)	.
Shepherd, 2007 TNT	CAD	Atorvastatin 80 mg Atorvastatin 10 mg	10001	5	61	81	15	131/78	65	2.5	1.5 (9.7) 0.1 (9.7)^bf^	.
Takazakura, 2015	DM	Atorvastatin 10 mg Pravastatin 10 mg Control	106	1	62	87	100	129/0	64	3.0	-0.80 (11.4) -2.80 (10.8) -3.10 (9.6)^a^	-0.2 (0.4) -0.1 (0.7) 0.1 (0.5)^a^ log(UACR)
Tonelli, 2005 PPP **	CAD	Pravastatin 40 mg Placebo	18569	5	58	90	7	133/81	73	4.2	Effect of pravastatin: 0.10 (0.02; 0.17) mL/min/1.73m^2 g^	.
Vidt, 2011 JUPITER	Healthy population	Rosuvastatin 20 mg Placebo	16279	2.3	66	62	31	.	75	.	-7.10 (11.9) -7.70 (11.8)	.
Yakusevich, 2013	Stroke	Simvastatin 40 mg Control	210	1	66	45	.	.	76	2.2	7.05 (12.1) 1.37 (13.8)^f^	.
Yasuda, 2004	CKD	Fluvastatin 20 mg Control	80	0.9	58	46	43	144/80	60	4.4	-8.67 (3.9) -6.50 (4.0)^a^	0 (0.14) 0 (0.15)^a^ (UAE)
De Zeeuw, 2015 PLANET I	DM	Rosuvastatin 10 mg Rosuvastatin 40 mg Atorvastatin 80 mg	325	1	58	70	100	139/79	71	3.9	-3.70 (14.7) -7.29 (20.4) -1.61 (13.0)	2 (79) -4 (77) -13 (57) %change
De Zeeuw, 2015 PLANET II	Non-DM proteinuria	Rosuvastatin 10 mg Rosuvastatin 40 mg Atorvastatin 80 mg	220	1	49	62	0	130/81	75	4.3	-2.71 (13.3) -3.30 (12.5) -1.74 (14.2)	-6 (99) 8 (75) -24 (60)

ACS, acute coronary syndrome; ADPKD, autosomal dominant polycystic kidney disease; CAD, coronary artery disease; CHD, coronary heart disease; CKD, chronic kidney disease; eGFR, estimated glomerular filtration rate; eze, ezetimibe; HT, hypertension; MI, myocardial infarction; TIA, transient ischemic attack; (NID−)DM1/DM2, non-insulin dependent diabetes mellitus 1 or 2, LDL, low-density lipoprotein; prot, proteinuria; UACR, urinary albumin-to-creatinine ratio; UAE, urinary albumin excretion; UPE, urinary protein excretion.

*Intervention was a combination of statin and vitamin E supplementation.

**PPP: Pravastatin Pooling Project, study representing pooled estimates of three RCTs: LIPID, CARE, and WOSCOPS. Individual data on each RCT was not published.

^a^based on eGFR (SD) value at baseline and follow-up. SD of eGFR change was calculated according to the formula provided in the Cochrane Handbook^11^.

^b^data extracted from figure.

^c^reported geometric mean was log-transformed to achieve normal distribution with symmetrical SD.

^d^SD acquired by dividing interquartile range by 1.35.

^e^no SD or SE reported, these were therefore borrowed from comparable studies.

^f^SD of baseline eGFR value used to calculate SD of eGFR change.

^g^only effect of treatment *vs* control reported.

### Pairwise comparison: statins and eGFR decline

**Figure 2:**
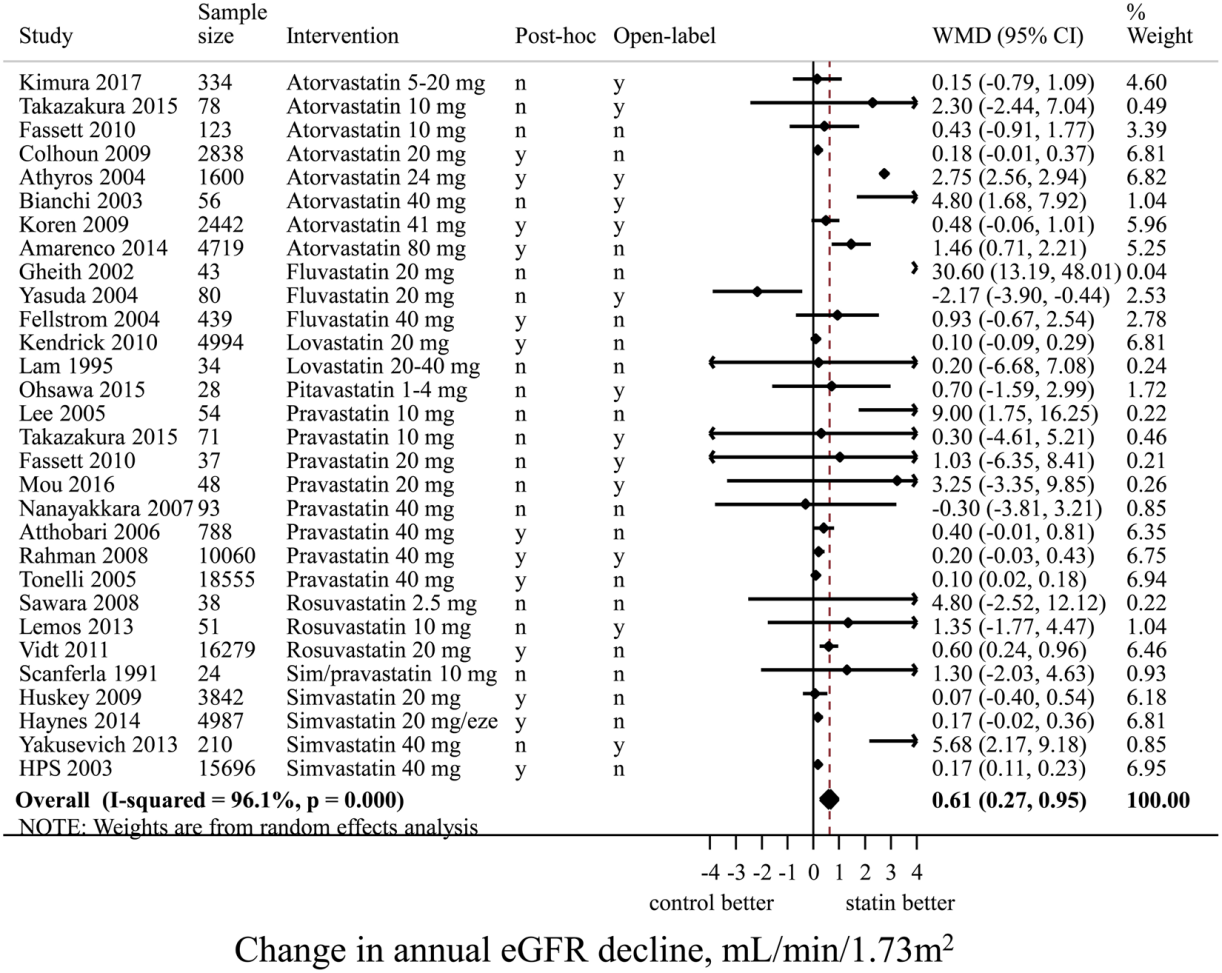
Pairwise random effects meta-analysis of randomized controlled trials investigating the effect of statin therapy versus control on the rate of annual eGFR decline. Positive values mean slower eGFR decline for statin users *vs* non-users, thus favouring statin use. In this forest plot, only 30 RCTs are included that compare a statin intervention vs non-statin control intervention. We thus excluded 13 RCTs that reported only the outcome proteinuria (n = 3), or that compared two statin interventions (n = 10). eGFR, estimated glomerular filtration rate; eze, ezetimibe 10 mg; CI, confidence interval; WMD, weighted mean difference; n, no; y, yes.

Except for two medium sized trials (Yasuda *et al*., and Nanayakkara *et al*.), effect estimates of all RCTs showed a protective effect of statin on eGFR decline^53,62^. Random-effects meta-analysis showed that statin use, compared to control, led to a 0.61 (95% CI 0.27; 0.95) mL/min/1.73 m^2^ slower annual eGFR decline (Fig. 2). When only RCTs with a sample size of at least 100 patients (n = 16) were analyzed, the beneficial effect of statin treatment on annual eGFR decline was 0.58 (95%-CI 0.23; 0.92) mL/min/1.73 m^2^. Heterogeneity between RCTs was high, with an I^2^ of 96%. Meta-regression showed that higher systolic blood pressure at baseline was significantly associated with smaller effects of statins, explaining 40% of the between-study variance. We found no evidence for interaction between diabetes and statins with regard to the beneficial effect on kidney function decline. Age, sex, serum LDL level, or change in LDL, had no significant impact on the effect estimates. In post-hoc RCTs (n = 11) the beneficial effect on annual kidney function decline of statins *vs* control was smaller but more precise than in RCTs in which change in eGFR was the primary outcome (n = 17): 0.55 (95%-CI 0.19; 0.92) *vs* 1.55 (95%-CI 0.26; 2.85) mL/min/1.73 m^2^, respectively. In open-label RCTs (n = 17, mean sample size 4326) the beneficial effect on eGFR decline of statins *vs* control was stronger than in blinded RCTs (n = 13, mean sample size 1161): 1.25 (95%-CI 0.08; 2.42) *vs* 0.23 (95%-CI 0.11; 0.34) mL/min/1.73 m^2^, respectively. The funnel plot for eGFR decline was slightly asymmetrical (Supplementary Figure S2), but the Egger’s test for small study effects was not significant (p = 0.3).

### Pairwise comparison: statins and proteinuria

**Figure 3:**
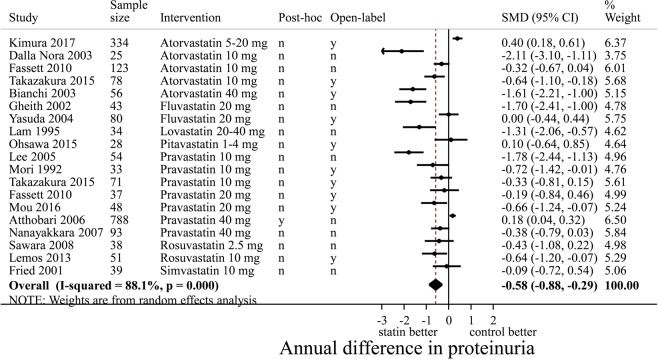
Pairwise random effects meta-analysis of randomized controlled trials investigating the effect of statin therapy versus control on the rate of annual change in proteinuria. Negative values mean a decrease in proteinuria for statin users *vs* non-users, thus favouring statin use. Effects expressed as SMD (standardized mean difference). In this forest plot, only 19 RCTs are included that compare a statin intervention vs non-statin control intervention. We thus excluded 24 RCTs that reported only the outcome proteinuria (n = 18), or that compared two statin interventions (n = 6). CI, confidence interval; SMD, standardized mean difference; n, no; y, yes.

The two largest RCTs showed that statin treatment *vs* control did not lower proteinuria: SMD of 0.40 (95%-CI 0.18; 0.61) and 0.18 (95%-CI 0.04; 0.32), respectively^32,63^. In a meta-analysis, statin use compared to control showed a significant reduction of proteinuria with an SMD −0.58 (95%-CI −0.88; −0.29) (Fig. 3). However, the funnel plot of the effect of statins on proteinuria suggested publication bias (Supplementary Figure S3) and the Egger’s test was significant (p < 0.001).

### Network meta-analysis

**Figure 4:**
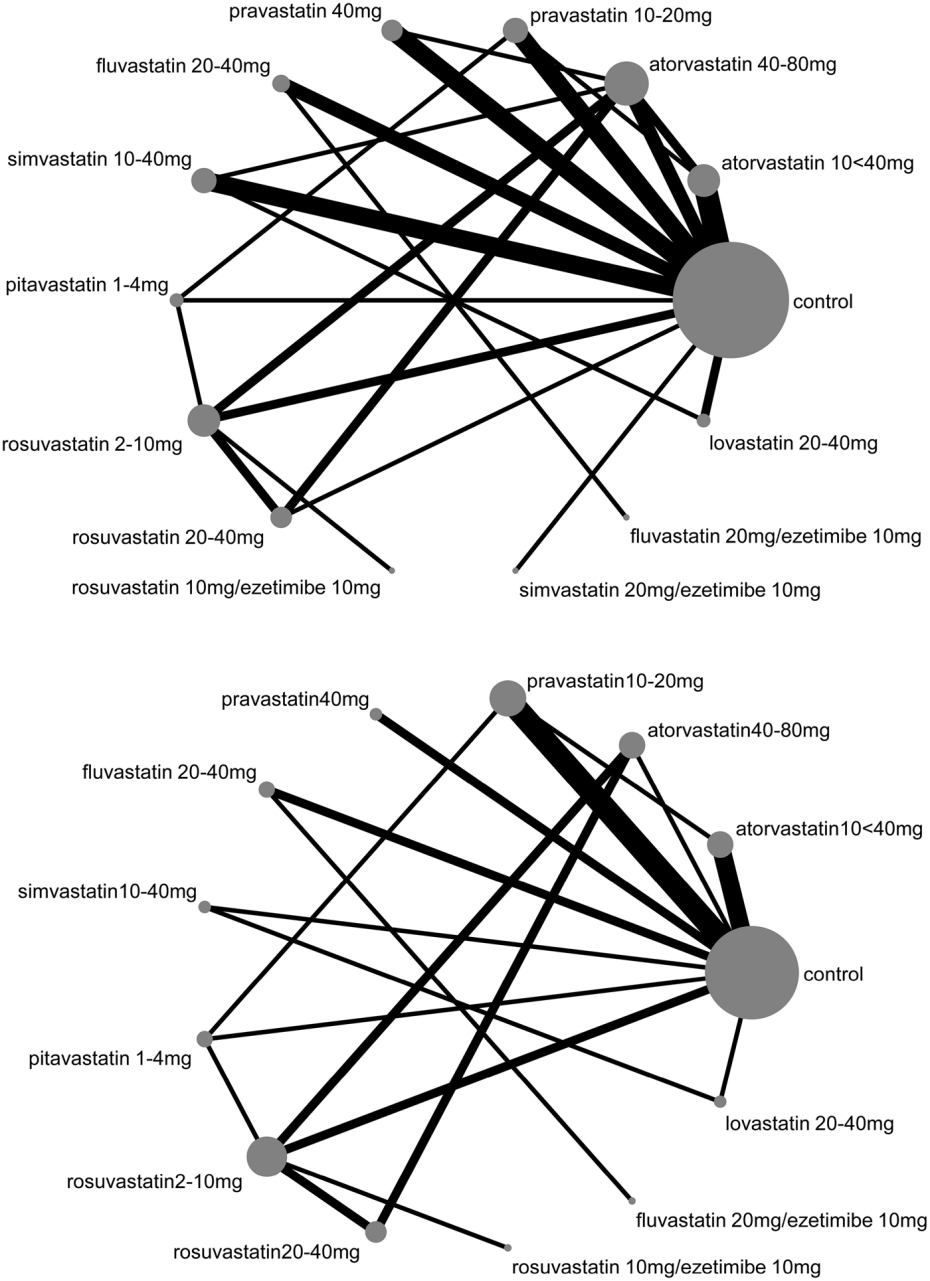
Network plots for the outcome eGFR decline (**A**) and proteinuria (**B**). The size of the nodes represents the number of RCTs for each treatment (ranging from 1 to 30; 30 for control intervention). The width of the connections represents the number of RCTs for each individual comparison (ranging from 1 to 5). eGFR, estimated glomerular filtration rate; RCT, randomized controlled trial.

**Figure 5:**
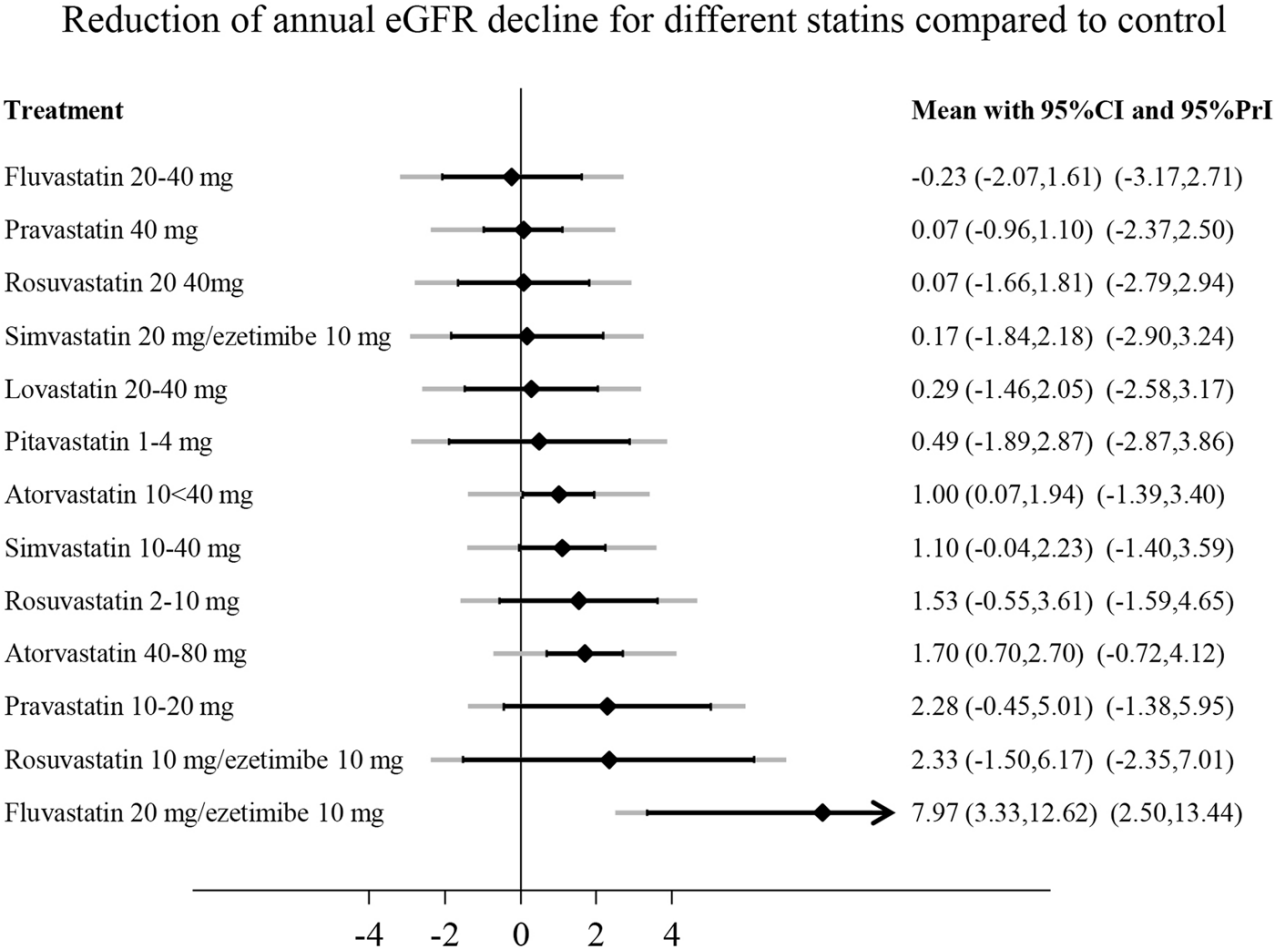
Effect of different statins compared to control treatment on annual eGFR decline. Effects are presented as weighted mean differences. Positive values represent a slower eGFR decline. Black lines around point estimates reflect 95%-confidence intervals and grey lines reflect prediction intervals. Prediction intervals represent the expected range of true effects of (future) similar studies and is suitable to assess the variability of an effect across different settings. CI, confidence interval; eGFR, estimated glomerular filtration rate; PrI, prediction interval.

Figure 4A shows the network plot of different statin treatments for change in eGFR. Each connection was formed by maximally 4 RCTs. We found no evidence for inconsistency in the network for eGFR decline and proteinuria using global tests (p-value for inconsistency 0.8) or local tests (p > 0.3 for all loops). We found that almost all statins performed better than control (Fig. 5). The most beneficial effect on eGFR decline was caused by fluvastatin 20 mg/ezetimibe 10 mg, rosuvastatin 20 mg/ezetimibe 10 mg, pravastatin 10–20 mg, and atorvastatin 40–80 and 10 < 40 mg. However, point estimates had broad 95%-confidence intervals and prediction intervals. Except for combined fluvastatin 20 mg/ezetimibe 10 mg and atorvastatin 40–80 mg, all 95%-confidence intervals crossed the line of no effect.

**Figure 6:**
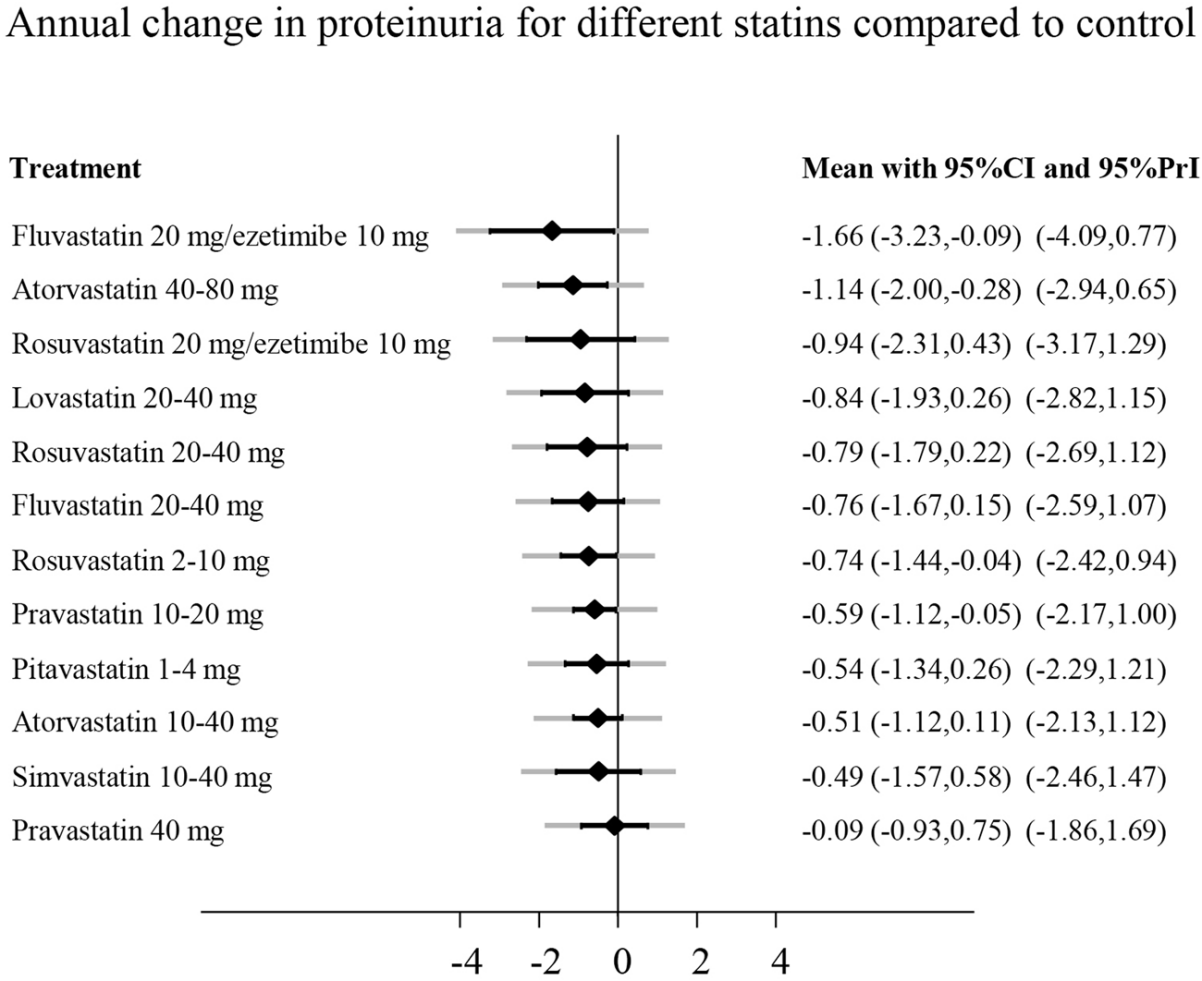
Effect of different statins compared to control treatment on annual change in proteinuria. Effects are presented as standardized mean differences (SMD). Negative values represent a reduction of proteinuria. Black lines around point estimates reflect 95%-confidence intervals and grey lines reflect prediction intervals. Prediction intervals represent the expected range of true effects in (future) similar studies and is suitable to assess the variability of effect across different settings. CI, confidence interval; eGFR, estimated glomerular filtration rate; PrI, prediction interval.

Figure 4B shows the network plot for all statin treatments regarding proteinuria. For proteinuria, no single RCT compared the combination therapy simvastatin/ezetimibe. Globally, there was no evidence for inconsistency (p-value 0.8). However, using local tests, there were 2 inconsistent loops: control, atorvastatin 40–80 mg, rosuvastatin 2–10 mg (p = 0.04) and control, simvastatin 10–40 mg, lovastatin 20–40 mg (p = 0.03). The inconsistencies between direct and indirect effects were introduced by the relatively large effect estimates of small studies (n < 60). The most efficacious treatments regarding proteinuria were fluvastatin 20 mg/ezetimibe 10 mg, atorvastatin 40–80 mg, and rosuvastatin 20 mg/ezetimibe 10 mg (Fig. 6).

**Figure 7:**
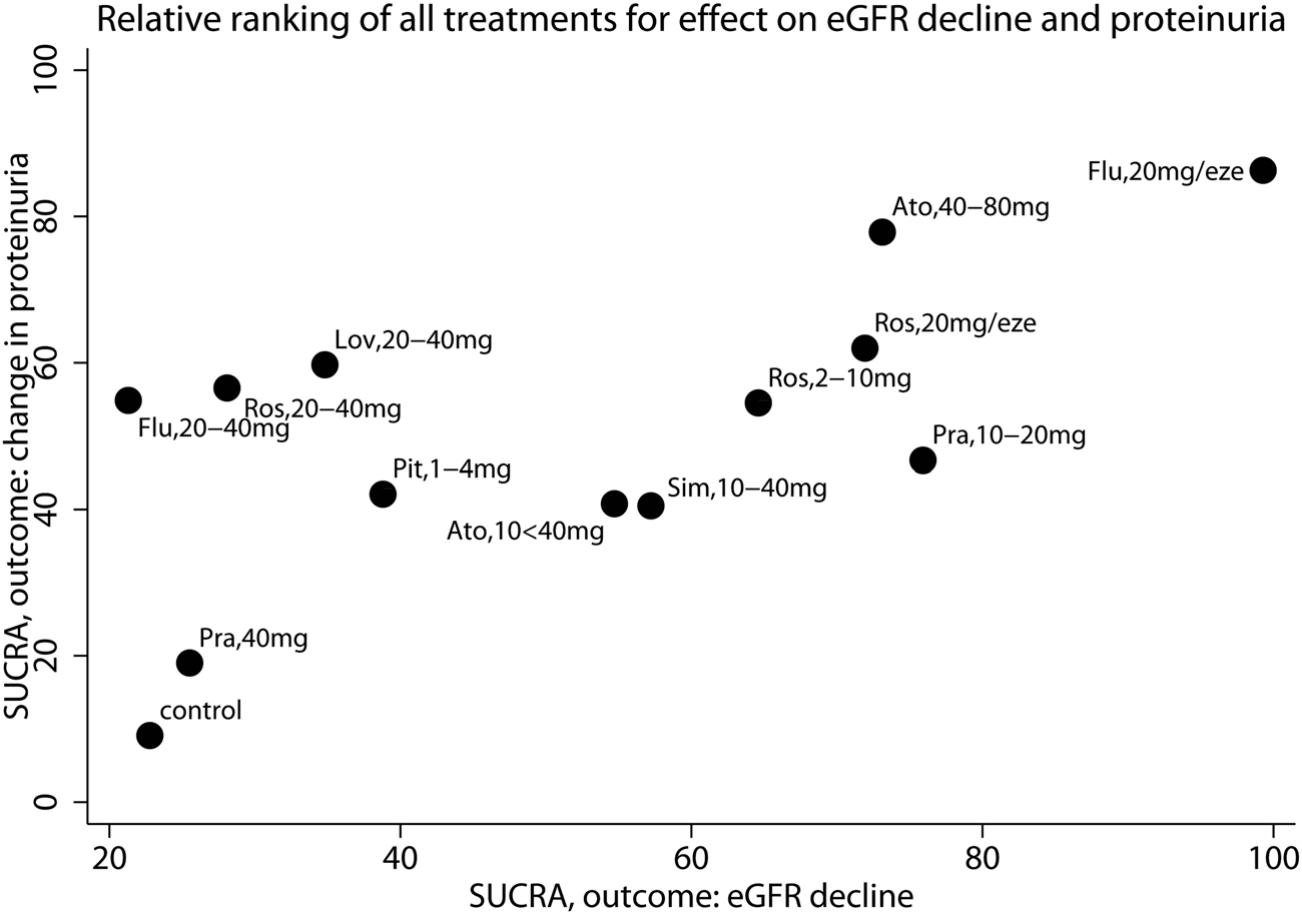
Each dot represents the SUCRA value of each treatment. The SUCRA takes into account for every treatment the cumulative probabilities of all possible rankings. If a treatment always ranks first or last, the SUCRA is 100% or 0%, respectively. The horizontal axis shows SUCRA values with regards to the outcome eGFR decline, the vertical axis shows the SUCRA for the outcome proteinuria. Ato, atorvastatin; eze, ezetimibe 10 mg; Flu, fluvastatin; Lov, lovastatin; Pit, pitavastatin; Pra, pravastatin; Ros, rosuvastatin; Sim, simvastatin; eGFR, estimated glomerular filtration rate; SUCRA, surface under the cumulative ranking curve.

Finally, SUCRA analysis showed that control treatment had the lowest SUCRA. Fluvastatin 20 mg/ezetimibe 10 mg had the highest SUCRA value for eGFR decline (99%) and fluvastatin 20 mg/ezetimibe 10 mg (86%) as well as atorvastatin 40–80 mg (78%) had the highest SUCRA value for change in proteinuria (Fig. 7).

### Sensitivity analyses

Since we included RCTs with seven different types of statin treatments with one or more different dosages, networks of subgroups had only few closed loops. Therefore, estimates were based mostly on either direct or indirect evidence, but not on mixed evidence. Nonetheless, we repeated the network meta-analysis for eGFR decline excluding RCTs with a sample size <100 (n = 16), excluding open-label RCTs (n = 17), or excluding post-hoc analyses (n = 20). Although effect estimates and rankings of individual treatments were variable across the analyses, in general atorvastatin 40–80 mg, fluvastatin 20 mg/ezetimibe 10 mg, pravastatin 10–20 mg, simvastatin 10–40 mg, and fluvastatin 20 mg were the most effective treatments with regard to eGFR decline. However, 95%-confidence intervals had substantial overlap, and individual treatments were rarely statistically significantly different from control. Since only a small number of RCTs with small sample sizes studied the effect of statins on proteinuria, we could not perform the aforementioned sensitivity analyses.

## Discussion

In this network meta-analysis, we showed that there are no substantial differences in the efficacy of seven different statins and dosages, with or without ezetimibe, regarding slowing down eGFR decline or reducing proteinuria. If anything, the combination of fluvastatin 20 mg/ezetimibe 10 mg and atorvastatin 40–80 mg most consistently had the strongest beneficial effect on both renal endpoints, but the differences between treatments were small and confidence intervals were wide. In the pairwise meta-analysis we showed that use of statins lowered the rate of annual kidney function decline by 0.61 mL/min/1.73 m^2^ and reduced the amount of proteinuria by −0.58 (95%-CI −0.88; −0.29) standard deviations per year.

Our results are in line with a recent meta-analysis Su *et al*. which reported that statins compared to control led to a 0.41 (95%-CI 0.11; 0.70) mL/min/1.73 m^2^ slower annual eGFR decline and a reduction of −0.65 (95%-CI −0.94; −0.37) standard deviations in proteinuria^8^. The small difference in outcomes between the present study and Su *et al*. are explained by different inclusion criteria. In contrast to the study of Su *et al*., we included three RCTs investigating combinations of statins plus ezetimibe. Including also treatments combining statins with ezetimibe, results in a more complete review of existing literature on lipid-lowering therapy by statins. As a consequence we incorporated in our meta-analysis three extra RCTs, including the SHARP trial (n = 5037). Furthermore, we excluded RCTs with a short follow-up (<12 months) or less than 10 patients per study arm, of which Su *et al*. included 19 RCTs. Finally, we found that the beneficial effect of statins on eGFR decline was weaker in RCTs with a higher mean systolic blood pressure. Systolic blood pressure explained 40% of the between-study variance. Taken together, these results suggest that a high systolic blood pressure modifies the effect of statins on eGFR decline. Hypertension is most likely a stronger risk factor for kidney function decline compared to hypercholesteremia. Therefore, we speculate that the positive effect of statins on kidney function decline is overwhelmed in the presence of high blood pressure.

In our network meta-analysis, we specifically investigated the efficacy of individual statins and different dosages, using both direct and indirect evidence. We showed that each different statin compared to placebo had a beneficial effect on the annual eGFR decline and reduced proteinuria. However, confidence intervals were broad for individual treatment comparisons in our network, due to the small number of RCTs contributing to each comparison. Su *et al*. showed in subgroup analyses the strongest beneficial effect on change in eGFR decline for atorvastatin, fluvastatin, and rosuvastatin^8^. However, they pooled for each statin all dosages. The validity of these comparisons may be limited, considering the clear differential effects of different dosages^8,66^.

We showed that fluvastatin 20 mg/ezetimibe 10 mg was the most efficacious treatment regarding both renal outcomes. However, this result was strongly influenced by the study of Kinouchi *et al*., comprising 54 patients, reporting an annual eGFR decline of −4.1 mL/min/1.73 m^2^ in patients treated with fluvastatin 20 mg compared to an annual eGFR increase of 4.1 mL/min/1.73 m^2^ in patients treated with fluvastatin 20 mg/ezetimibe 10 mg^46^. Since the average annual eGFR decline in adults with a history of cardiovascular disease is about 2 mL/min/1.73 m^2^, the reported effect of Kinouchi *et al*. of 8.2 mL/min/1.73 m^2^ is large, and should be interpreted with caution^67^. We found that the second most efficacious statin on both renal endpoints was high dose atorvastatin, which improved the annual eGFR decline by 1.70 (95%-CI 0.70; 2.70) mL/min/1.73 m^2^ and reduced proteinuria by 1.14 (95%-CI 0.28; 2.00) standard deviations, compared to control.

Statins included in the present study reduced LDL levels on average by 27%, which is in line with a previous meta-analysis showing an LDL-lowering effect for all statins^66^. However, there is no clear evidence that high LDL itself increases CKD risk^68^. Statins also may have pleiotropic effects favourable for reducing CKD progression, such as lowering oxidative stress, reducing inflammation, and stabilizing atherosclerotic plaques^7,69^. Hence, current guidelines recommend a statin for patients at risk for CKD, independent of LDL levels^9,70^.

The main strength of the current study is that we performed a network meta-analysis, in addition to a pairwise meta-analysis, to investigate differential effects of different statins with or without ezetimibe. We only included RCTs because they are more likely to provide unbiased information. We excluded small trials (<10 patients per arm) since they are more susceptible to publication bias.

This network meta-analysis has several limitations. First, heterogeneity was high (I^2^ = 96%) owing to variation of the included patient populations across RCTs, differences in blinding methods, randomization procedures, sample size, and variability in primary endpoints. The I^2^ statistic represents statistical heterogeneity, rather than clinically relevant heterogeneity, and is most strongly affected by the sample size of the individual studies. Upon increasing precision (sample size) of studies within a meta-analysis, the I^2^ statistic rapidly approaches 100%^71^. Deciding whether it is valid to pool studies, should be based on the clinical relevance of any present heterogeneity, rather than solely on the I^2^ statistic^71^. We used random effects models to take heterogeneity into account. Second, we found an asymmetric funnel plot regarding proteinuria, which may be an indication of publication bias. On the other hand, larger compared to smaller RCTs showed a weak but opposite effect. Thus, the asymmetry may also be the consequence of inclusion of smaller RCTs with lower quality. Therefore, we cannot rule out that the beneficial effect of statins on proteinuria is an overestimation. Additionally, there were relatively few RCTs investigating the effect of statins on proteinuria, and most of them were small (sample size <100). Small studies therefore had a large impact on the network meta-analysis estimates, introducing inconsistencies especially in loops comprising small numbers of RCTs. The advantage of a network analysis is that it takes both direct and indirect effects into account, reducing the impact of single studies with a small sample size. For the outcome eGFR decline, the sample sizes of the included RCTs were large (24 RCTs with n >100) which improved precision and reduced potential publication bias. The much smaller effect of statins compared to control in double blind compared to open-label RCTs may suggest bias due to the lack of blinding in the open-label RCTs. Since 17 out of 30 RCTs were open-label, we may have overestimated the beneficial effect on eGFR decline of statins compared to control. Third, due to the low number of RCTs contributing to each connection in the network meta-analyses, there was insufficient power to detect differences between statins. Fourth, a large number of the included RCTs used the MDRD formula to estimate eGFR, which is known to underestimate the true eGFR for values reported higher than 60 mL/min/1.73 m^2^ ^72^. If anything, this may have underestimated the beneficial effect of statin use compared to control in studies with a mean eGFR higher than 60 mL/min/1.73 m^2^.

In conclusion, we found a beneficial effect of different statins, with or without ezetimibe, compared to control on progression of eGFR decline, and possibly proteinuria. Due to the imprecision of individual comparisons, results were inconclusive as to which statin performs best with regard to renal outcome.

## Supplementary information

Supplementary data
