# Supplementary material for: Effect of different types of statins on kidney function decline and proteinuria: a network meta-analysis
Source: Sci Rep. 2019 Nov 12;9:16632. doi: 10.1038/s41598-019-53064-x (PMC6851118; doi:10.1038/s41598-019-53064-x)
Supplement: Supplementary file 1 — Supplementary data [file 41598_2019_53064_MOESM1_ESM.pdf]

Effect of different types of statins on kidney function decline and proteinuria: a network meta-analysis

K. Esmeijer[1, 2], Olaf M. Dekkers[2, 3], Johan W. de Fijter[1], Friedo W. Dekker[2], Ellen K. Hoogeveen[1, 2, 4]

[1] Department of Nephrology, Leiden University Medical Center, Leiden, The Netherlands

[2] Department of Clinical Epidemiology, Leiden University Medical Center, Leiden, The Netherlands

[3] Department of Endocrinology, Leiden University Medical Center, Leiden, The Netherlands

[4] Department of Nephrology, Jeroen Bosch Hospital, Den Bosch, The Netherlands

Supplemental Data

Supplementary Figure S1: Risk of bias assessment per study (left panel) and summarized over all studies (right panel), according to the Cochrane Risk of Bias tool. Red, green and yellow cells mean high, low, and unclear risk of bias, respectively. Pl I and Pl II refer to PLANET I and II trials, respectively.

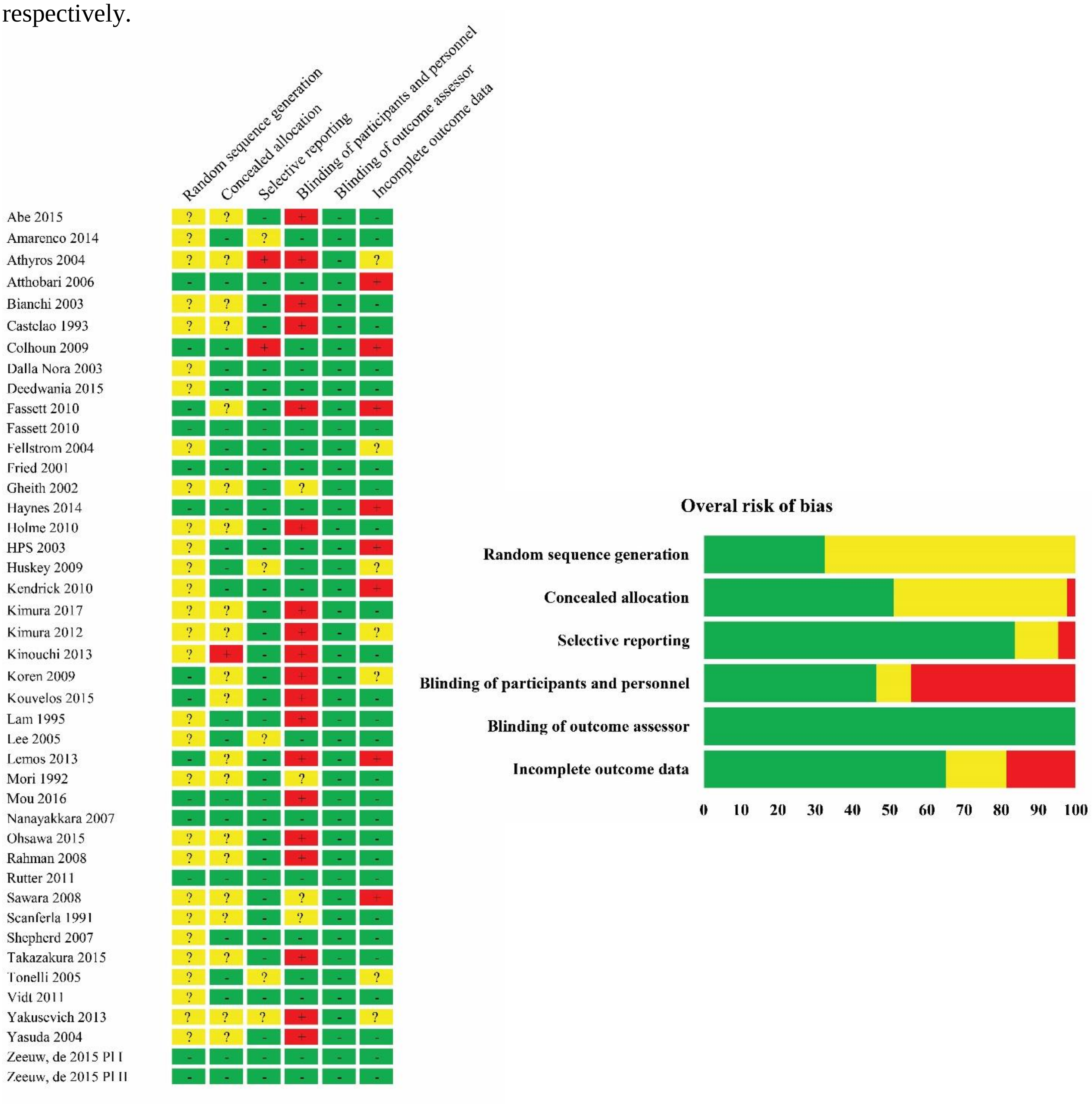

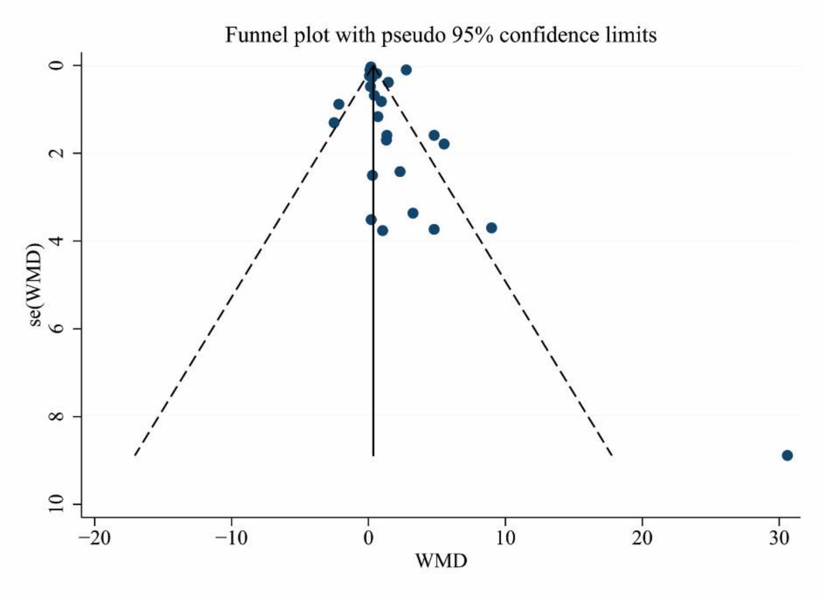

Supplementary Figure S2: Funnel plot of included randomized controlled trials investigating the effect of statin therapy on annual eGFR decline. According to Egger's test there was no evidence for publication bias (p = 0.3).

eGFR, estimated glomerular filtration rate; WMD, weighted mean difference.

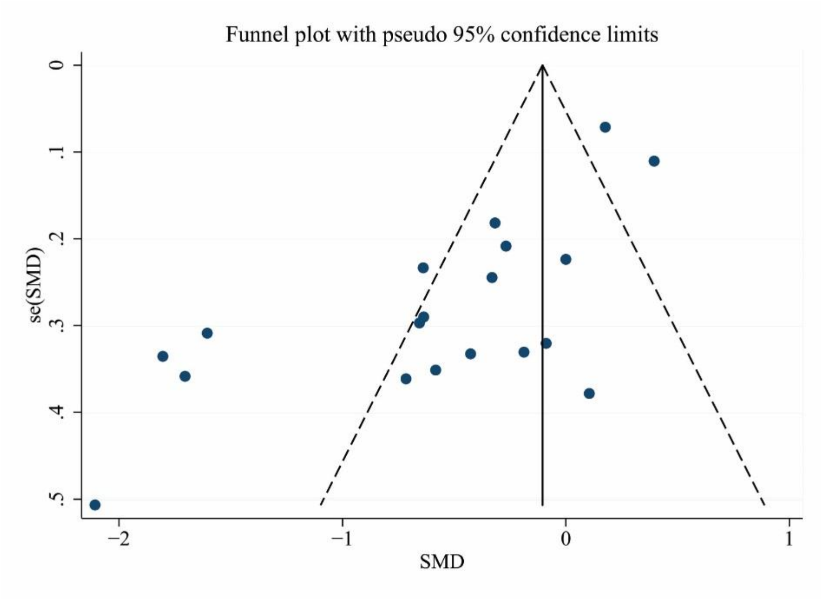

Supplementary Figure S3: Funnel plot of included randomized controlled trials investigating the effect of statin therapy on change in proteinura. According to Egger's test there was significant evidence for publication bias (p <0.001).

SMD, standardized mean difference

Appendix

Search strategy

PubMed

(("Hydroxymethylglutaryl-CoA Reductase Inhibitors"[Mesh] OR "Hydroxymethylglutaryl-CoA Reductase Inhibitors"[Pharmacological Action] OR "Hydroxymethylglutaryl-CoA Reductase Inhibitors"[tw] OR "Hydroxymethylglutaryl-CoA Reductase Inhibitor"[tw] OR "HMG-CoA Reductase Inhibitors"[tw] OR "HMG-CoA Reductase Inhibitor"[tw] OR "HMG CoA Reductase Inhibitors"[tw] OR "HMG CoA Reductase Inhibitor"[tw] OR "Statins"[tw] OR "Statin"[tw] OR "Hydroxymethylglutaryl-CoA Inhibitors"[tw] OR "Hydroxymethylglutaryl-CoA Inhibitor"[tw] OR "Hydroxymethylglutaryl-Coenzyme A Inhibitors"[tw] OR "Hydroxymethylglutaryl-Coenzyme A Inhibitor"[tw] OR "2,6,7-trideoxy-7-C-(2,4-dichlorophenyl)heptonic acid"[Supplementary Concept] OR "2-(2-(3-hydroxy-12-(2-methyl-1-oxobutoxy)-5-androstan-17-yl)ethyl)tetrahydro-4-hydroxy-2H-pyran-6-one"[Supplementary Concept] OR "3,24-dihydroxycholest-8(14)-en-15-one"[Supplementary Concept] OR "3,25-dihydroxycholest-8(14)-en-15-one"[Supplementary Concept] OR "3,4-dihydroxyphenylpropionylleucinamide"[Supplementary Concept] OR "3-hydroxy-24-dimethylaminochol-8(14)-en-15-one"[Supplementary Concept] OR "3-hydroxy-25,26,26,26,27,27,27-heptafluoro-5-cholest-8(14)-en-15-one"[Supplementary Concept] OR "3-hydroxycholest-8(14),24-dien-15-one"[Supplementary Concept] OR "6-(2,4-dichlorophenyl)-2,3,4-trihydroxyhexanesulfonic acid"[Supplementary Concept] OR "6-(2,4-dichlorophenyl)-2,4-dihydroxyhexane-1-sulfonic acid"[Supplementary Concept] OR "6-(2,4-dichlorophenyl)-erythro-2,4-dihydroxyhexylphosphonic acid"[Supplementary Concept] OR "6-(2-(2,4-dichloro-6-hydroxyphenyl)ethyl)-3,4,5,6-tetrahydro-4-hydroxy-2H-pyran-2-one"[Supplementary Concept] OR "6-(2-(8-((4-fluorobenzyl)oxy)-1,2,6,7,8,8a-hexahydro-2,6-dimethyl-1-naphthyl)ethyl)-4-hydroxy-3,4,5,6-tetrahydro-2H-pyran-2-one"[Supplementary Concept] OR "6-hydroxyisocompactin"[Supplementary Concept] OR "7-(2'-((2"-methyl-1"-oxobutoxy)methyl)-1'-cyclohexyl)-3-hydroxy-5-oxoheptanoic acid"[Supplementary Concept] OR "7-(2,4-dichlorophenyl)-3-hydroxy-5-heptanolide"[Supplementary Concept] OR "7-(3-bromo-4,5-bis(4-fluorophenyl)-2-(1-methylethyl)-1H-pyrrol-1-yl)-3,5-dihydroxy-6-heptenoic acid methyl ester"[Supplementary Concept] OR "7-(3-hydroxy-2-methyl-1,2,3,5,6,7,8,8a-octahydro-1-naphtalenyl)-3,5-dihydroxyheptanoic acid"[Supplementary Concept] OR "7-(7-((1,1'-biphenyl-4-yl)methyl)-2-(4-fluorophenyl)-4,5,6,7-tetrahydro-2H-indazol-3-yl)-3,5-dihydroxy-6-heptenoic acid"[Supplementary Concept] OR "amlodipine, atorvastatin drug combination"[Supplementary Concept] OR "Atorvastatin Calcium"[mesh] OR "BMY 21950"[Supplementary Concept] OR "cerivastatin"[Supplementary Concept] OR "cholest-8-ene-3,15-diol"[Supplementary Concept] OR "chrysosporin"[Supplementary Concept] OR "crilvastatin"[Supplementary Concept] OR "dihydrocompactin"[Supplementary Concept] OR "dihydromevinolin"[Supplementary Concept] OR "fermodulin"[Supplementary Concept] OR "fluvastatin"[Supplementary Concept] OR "GR 92549"[Supplementary Concept] OR "GR 95030X"[Supplementary Concept] OR "HR 780"[Supplementary Concept] OR "L 157012"[Supplementary Concept] OR "L 645164"[Supplementary Concept] OR "L 647318"[Supplementary Concept] OR "L 669262"[Supplementary Concept] OR "lanost-8-en-3-ol-24-one"[Supplementary Concept] OR "Lovastatin"[mesh] OR "Meglutol"[mesh] OR "methoxypolyethoxylated cholesterol"[Supplementary Concept] OR "mevastatin"[Supplementary Concept] OR "pannorin"[Supplementary Concept] OR "PD 123244-15"[Supplementary Concept] OR "phosphoadenosine diphosphoribose"[Supplementary

Concept] OR "pitavastatin"[Supplementary Concept] OR "Pravastatin"[mesh] OR "red yeast rice"[Supplementary Concept] OR "Rosuvastatin Calcium"[mesh] OR "RP 61969"[Supplementary Concept] OR "Simvastatin"[mesh] OR "SQ 33600"[Supplementary Concept] OR "SRI 62320"[Supplementary Concept] OR "2,6,7-trideoxy-7-C-(2,4-dichlorophenyl)heptonic acid"[tw] OR "2-(2-(3-hydroxy-12-(2-methyl-1-oxobutoxy)-5-androstan-17-yl)ethyl)tetrahydro-4-hydroxy-2H-pyran-6-one"[tw] OR "3,24-dihydroxycholest-8(14)-en-15-one"[tw] OR "3,25-dihydroxycholest-8(14)-en-15-one"[tw] OR "3,4-dihydroxyphenylpropionylleucinamide"[tw] OR "3-hydroxy-24-dimethylaminochol-8(14)-en-15-one"[tw] OR "3-hydroxy-25,26,26,26,27,27,27-heptafluoro-5-cholest-8(14)-en-15-one"[tw] OR "3-hydroxycholest-8(14),24-dien-15-one"[tw] OR "6-(2,4-dichlorophenyl)-2,3,4-trihydroxyhexanesulfonic acid"[tw] OR "6-(2,4-dichlorophenyl)-2,4-dihydroxyhexane-1-sulfonic acid"[tw] OR "6-(2,4-dichlorophenyl)-erythro-2,4-dihydroxyhexylphosphonic acid"[tw] OR "6-(2-(2,4-dichloro-6-hydroxyphenyl)ethyl)-3,4,5,6-tetrahydro-4-hydroxy-2H-pyran-2-one"[tw] OR "6-(2-(8-((4-fluorobenzyl)oxy)-1,2,6,7,8,8a-hexahydro-2,6-dimethyl-1-naphthyl)ethyl)-4-hydroxy-3,4,5,6-tetrahydro-2H-pyran-2-one"[tw] OR "6-hydroxyisocompactin"[tw] OR "7-(2'-((2"-methyl-1"-oxobutoxy)methyl)-1'-cyclohexyl)-3-hydroxy-5-oxoheptanoic acid"[tw] OR "7-(2,4-dichlorophenyl)-3-hydroxy-5-heptanolide"[tw] OR "7-(3-bromo-4,5-bis(4-fluorophenyl)-2-(1-methylethyl)-1H-pyrrol-1-yl)-3,5-dihydroxy-6-heptenoic acid methyl ester"[tw] OR "7-(3-hydroxy-2-methyl-1,2,3,5,6,7,8,8a-octahydro-1-naphtalenyl)-3,5-dihydroxyheptanoic acid"[tw] OR "7-(7-((1,1'-biphenyl-4-yl)methyl)-2-(4-fluorophenyl)-4,5,6,7-tetrahydro-2H-indazol-3-yl)-3,5-dihydroxy-6-heptenoic acid"[tw] OR "amlodipine, atorvastatin drug combination"[tw] OR "Atorvastatin"[tw] OR "BMY 21950"[tw] OR "cerivastatin"[tw] OR "cholest-8-ene-3,15-diol"[tw] OR "chrysosporin"[tw] OR "crilvastatin"[tw] OR "dihydrocompactin"[tw] OR "dihydromevinolin"[tw] OR "fermodulin"[tw] OR "fluvastatin"[tw] OR "GR 92549"[tw] OR "GR 95030X"[tw] OR "HR 780"[tw] OR "L 157012"[tw] OR "L 645164"[tw] OR "L 647318"[tw] OR "L 669262"[tw] OR "lanost-8-en-3-ol-24-one"[tw] OR "Lovastatin"[tw] OR "Meglutol"[tw] OR "methoxypolyethoxylated cholesterol"[tw] OR "mevastatin"[tw] OR "pannorin"[tw] OR "PD 123244-15"[tw] OR "phosphoadenosine diphosphoribose"[tw] OR "pitavastatin"[tw] OR "Pravastatin"[tw] OR "red yeast rice"[tw] OR "Rosuvastatin Calcium"[tw] OR "RP 61969"[tw] OR "Simvastatin"[tw] OR "SQ 33600"[tw] OR "SRI 62320"[tw]) AND ("renal dysfunction"[tw] OR renal dysfunction*[tw] OR "kidney dysfunction"[tw] OR kidney dysfunction*[tw] OR "kidney function loss"[tw] OR "renal function loss"[tw] OR "kidney function"[tw] OR kidney function*[tw] OR "renal function"[tw] OR renal function*[tw] OR "Renal Insufficiency, Chronic"[Mesh] OR "Chronic Kidney Disease"[tw] OR "Chronic Renal Disease"[tw] OR "Kidney Failure"[tw] OR "Renal Failure"[tw] OR "Proteinuria"[Mesh:noexp] OR "Proteinuria"[tw] OR "Albuminuria"[mesh] OR "Albuminuria"[tw] OR "macroalbuminuria"[tw] OR "macro-albuminuria"[tw] OR "microalbuminuria"[tw] OR "micro-albuminuria"[tw] OR "urinary protein"[tw] OR "urinary proteins"[tw] OR "urinary albumin"[tw] OR "Glomerular Filtration Rate"[mesh] OR "Glomerular Filtration Rate"[tw] OR "eGFR"[tw] OR "e-GFR"[tw] OR "GFR"[tw] **OR** "Kidney/drug effects"[mesh] OR "Kidney/physiopathology"[mesh] OR "Renal Insufficiency"[mesh] OR "Kidney Function Tests"[mesh]) AND ("Clinical Trial"[Publication Type] OR "trial"[ti] OR "rct"[tw] OR "randomized"[ti] OR "randomised"[ti])) NOT (("meta-analysis"[ptyp] OR "systematic"[sb]) NOT "Clinical Trial"[ptyp]))

Embase

((exp *"hydroxymethylglutaryl coenzyme A reductase inhibitor"/ OR "Hydroxymethylglutaryl-CoA Reductase Inhibitors".ti,ab OR "Hydroxymethylglutaryl-CoA Reductase Inhibitor".ti,ab OR "HMG-CoA Reductase Inhibitors".ti,ab OR "HMG-CoA Reductase Inhibitor".ti,ab OR "HMG CoA Reductase Inhibitors".ti,ab OR "HMG CoA Reductase Inhibitor".ti,ab OR "Statins".ti,ab OR "Statin".ti,ab OR "Hydroxymethylglutaryl-CoA Inhibitors".ti,ab OR "Hydroxymethylglutaryl-CoA Inhibitor".ti,ab OR "Hydroxymethylglutaryl-Coenzyme A Inhibitors".ti,ab OR "Hydroxymethylglutaryl-Coenzyme A Inhibitor".ti,ab OR "2,6,7-trideoxy-7-C-(2,4-dichlorophenyl)heptonic acid".ti,ab OR "2-(2-(3-hydroxy-12-(2-methyl-1-oxobutoxy)-5-androstan-17-yl)ethyl)tetrahydro-4-hydroxy-2H-pyran-6-one".ti,ab OR "3,24-dihydroxycholest-8(14)-en-15-one".ti,ab OR "3,25-dihydroxycholest-8(14)-en-15-one".ti,ab OR "3,4-dihydroxyphenylpropionylleucinamide".ti,ab OR "3-hydroxy-24-dimethylaminochol-8(14)-en-15-one".ti,ab OR "3-hydroxy-25,26,26,26,27,27,27-heptafluoro-5-cholest-8(14)-en-15-one".ti,ab OR "3-hydroxycholest-8(14),24-dien-15-one".ti,ab OR "6-(2,4-dichlorophenyl)-2,3,4-trihydroxyhexanesulfonic acid".ti,ab OR "6-(2,4-dichlorophenyl)-2,4-dihydroxyhexane-1-sulfonic acid".ti,ab OR "6-(2,4-dichlorophenyl)-erythro-2,4-dihydroxyhexylphosphonic acid".ti,ab OR "6-(2-(2,4-dichloro-6-hydroxyphenyl)ethyl)-3,4,5,6-tetrahydro-4-hydroxy-2H-pyran-2-one".ti,ab OR "6-(2-(8-((4-fluorobenzyl)oxy)-1,2,6,7,8,8a-hexahydro-2,6-dimethyl-1-naphthyl)ethyl)-4-hydroxy-3,4,5,6-tetrahydro-2H-pyran-2-one".ti,ab OR "6-hydroxyisocompactin".ti,ab OR "7-(2'-((2"-methyl-1"-oxobutoxy)methyl)-1'-cyclohexyl)-3-hydroxy-5-oxoheptanoic acid".ti,ab OR "7-(2,4-dichlorophenyl)-3-hydroxy-5-heptanolide".ti,ab OR "7-(3-bromo-4,5-bis(4-fluorophenyl)-2-(1-methylethyl)-1H-pyrrol-1-yl)-3,5-dihydroxy-6-heptenoic acid methyl ester".ti,ab OR "7-(3-hydroxy-2-methyl-1,2,3,5,6,7,8,8a-octahydro-1-naphtalenyl)-3,5-dihydroxyheptanoic acid".ti,ab OR "7-(7-((1,1'-biphenyl-4-yl)methyl)-2-(4-fluorophenyl)-4,5,6,7-tetrahydro-2H-indazol-3-yl)-3,5-dihydroxy-6-heptenoic acid".ti,ab OR "amlodipine, atorvastatin drug combination".ti,ab OR "Atorvastatin".ti,ab OR "BMY 21950".ti,ab OR "cerivastatin".ti,ab OR "cholest-8-ene-3,15-diol".ti,ab OR "chrysosporin".ti,ab OR "crilvastatin".ti,ab OR "dihydrocompactin".ti,ab OR "dihydromevinolin".ti,ab OR "fermodulin".ti,ab OR "fluvastatin".ti,ab OR "GR 92549".ti,ab OR "GR 95030X".ti,ab OR "HR 780".ti,ab OR "L 157012".ti,ab OR "L 645164".ti,ab OR "L 647318".ti,ab OR "L 669262".ti,ab OR "lanost-8-en-3-ol-24-one".ti,ab OR "Lovastatin".ti,ab OR "Meglutol".ti,ab OR "methoxypolyethoxylated cholesterol".ti,ab OR "mevastatin".ti,ab OR "pannorin".ti,ab OR "PD 123244-15".ti,ab OR "phosphoadenosine diphosphoribose".ti,ab OR "pitavastatin".ti,ab OR "Pravastatin".ti,ab OR "red yeast rice".ti,ab OR "Rosuvastatin Calcium".ti,ab OR "RP 61969".ti,ab OR "Simvastatin".ti,ab OR "SQ 33600".ti,ab OR "SRI 62320".ti,ab) AND (exp *"Kidney Dysfunction"/ OR "renal dysfunction".ti,ab OR renal dysfunction*.ti,ab OR "kidney dysfunction".ti,ab OR kidney dysfunction*.ti,ab OR "kidney function loss".ti,ab OR "renal function loss".ti,ab OR "kidney function".ti,ab OR kidney function*.ti,ab OR "renal function".ti,ab OR renal function*.ti,ab OR exp *"Kidney Failure"/ OR "Chronic Kidney Disease".ti,ab OR "Chronic Renal Disease".ti,ab OR "Kidney Failure".ti,ab OR "Renal Failure".ti,ab OR exp *"Proteinuria"/ OR "Proteinuria".ti,ab OR exp *"Albuminuria"/ OR "Albuminuria".ti,ab OR "macroalbuminuria".ti,ab OR "macro-albuminuria".ti,ab OR "microalbuminuria".ti,ab OR "micro-albuminuria".ti,ab OR "urinary protein".ti,ab OR "urinary proteins".ti,ab OR "urinary albumin".ti,ab OR exp *"Glomerulus Filtration Rate"/ OR "Glomerular Filtration Rate".ti,ab OR "eGFR".ti,ab OR "e-GFR".ti,ab OR "GFR".ti,ab OR *"Kidney Function Test"/) AND (exp "Clinical Trial"/ OR "trial".ti OR "rct".ti,ab OR "randomized".ti OR "randomised".ti) NOT ((exp "meta-analysis"/ OR "systematic review"/) NOT exp "Clinical Trial"/))

- NOT conference review.pt
- NOT (conference review or conference abstract).pt
- AND (conference abstract).pt

Web of Science

(ti=("hydroxymethylglutaryl coenzyme A reductase inhibitor" OR "Hydroxymethylglutaryl-CoA Reductase Inhibitors" OR "Hydroxymethylglutaryl-CoA Reductase Inhibitor" OR "HMG-CoA Reductase Inhibitors" OR "HMG-CoA Reductase Inhibitor" OR "HMG CoA Reductase Inhibitors" OR "HMG CoA Reductase Inhibitor" OR "Statins" OR "Statin" OR "Hydroxymethylglutaryl-CoA Inhibitors" OR "Hydroxymethylglutaryl-CoA Inhibitor" OR "Hydroxymethylglutaryl-Coenzyme A Inhibitors" OR "Hydroxymethylglutaryl-Coenzyme A Inhibitor" OR "2,6,7-trideoxy-7-C-(2,4-dichlorophenyl)heptonic acid" OR "2-(2-(3-hydroxy-12-(2-methyl-1-oxobutoxy)-5-androstan-17-yl)ethyl)tetrahydro-4-hydroxy-2H-pyran-6-one" OR "3,24-dihydroxycholest-8(14)-en-15-one" OR "3,25-dihydroxycholest-8(14)-en-15-one" OR "3,4-dihydroxyphenylpropionylleucinamide" OR "3-hydroxy-24-dimethylaminochol-8(14)-en-15-one" OR "3-hydroxy-25,26,26,26,27,27,27-heptafluoro-5-cholest-8(14)-en-15-one" OR "3-hydroxycholest-8(14),24-dien-15-one" OR "6-(2,4-dichlorophenyl)-2,3,4-trihydroxyhexanesulfonic acid" OR "6-(2,4-dichlorophenyl)-2,4-dihydroxyhexane-1-sulfonic acid" OR "6-(2,4-dichlorophenyl)-erythro-2,4-dihydroxyhexylphosphonic acid" OR "6-(2-(2,4-dichloro-6-hydroxyphenyl)ethyl)-3,4,5,6-tetrahydro-4-hydroxy-2H-pyran-2-one" OR "6-(2-(8-((4-fluorobenzyl)oxy)-1,2,6,7,8,8a-hexahydro-2,6-dimethyl-1-naphthyl)ethyl)-4-hydroxy-3,4,5,6-tetrahydro-2H-pyran-2-one" OR "6-hydroxyisocompactin" OR "7-(2'-((2"-methyl-1"-oxobutoxy)methyl)-1'-cyclohexyl)-3-hydroxy-5-oxoheptanoic acid" OR "7-(2,4-dichlorophenyl)-3-hydroxy-5-heptanolide" OR "7-(3-bromo-4,5-bis(4-fluorophenyl)-2-(1-methylethyl)-1H-pyrrol-1-yl)-3,5-dihydroxy-6-heptenoic acid methyl ester" OR "7-(3-hydroxy-2-methyl-1,2,3,5,6,7,8,8a-octahydro-1-naphtalenyl)-3,5-dihydroxyheptanoic acid" OR "7-(7-((1,1'-biphenyl-4-yl)methyl)-2-(4-fluorophenyl)-4,5,6,7-tetrahydro-2H-indazol-3-yl)-3,5-dihydroxy-6-heptenoic acid" OR "amlodipine, atorvastatin drug combination" OR "Atorvastatin" OR "BMY 21950" OR "cerivastatin" OR "cholest-8-ene-3,15-diol" OR "chrysosporin" OR "crilvastatin" OR "dihydrocompactin" OR "dihydromevinolin" OR "fermodulin" OR "fluvastatin" OR "GR 92549" OR "GR 95030X" OR "HR 780" OR "L 157012" OR "L 645164" OR "L 647318" OR "L 669262" OR "lanost-8-en-3-ol-24-one" OR "Lovastatin" OR "Meglutol" OR "methoxypolyethoxylated cholesterol" OR "mevastatin" OR "pannorin" OR "PD 123244-15" OR "phosphoadenosine diphosphoribose" OR "pitavastatin" OR "Pravastatin" OR "red yeast rice" OR "Rosuvastatin Calcium" OR "RP 61969" OR "Simvastatin" OR "SQ 33600" OR "SRI 62320") AND ti=("Kidney Dysfunction" OR "renal dysfunction" OR "renal dysfunction*" OR "kidney dysfunction" OR "kidney dysfunction*" OR "kidney function loss" OR "renal function loss" OR "kidney function" OR "kidney function*" OR "renal function" OR "renal function*" OR "Kidney Failure" OR "Chronic Kidney Disease" OR "Chronic Renal Disease" OR "Kidney Failure" OR "Renal Failure" OR "Proteinuria" OR "Proteinuria" OR "Albuminuria" OR "Albuminuria" OR "macroalbuminuria" OR "macro-albuminuria" OR "microalbuminuria" OR "micro-albuminuria" OR "urinary protein" OR "urinary proteins" OR "urinary albumin" OR "Glomerulus Filtration Rate" OR "Glomerular Filtration Rate" OR "eGFR" OR "e-GFR" OR "GFR" OR "Kidney Function Test") AND ts=("Clinical Trial" OR "trial" OR "rct" OR "randomized" OR "randomised") NOT ti=(("meta-analysis" OR "systematic review") NOT ("Trial" OR "rct" OR "randomized" OR "randomised")) NOT ti=(veterinary OR rabbit OR rabbits OR animal OR animals OR mouse OR mice OR rodent OR rodents OR rat OR rats OR pig OR pigs OR porcine OR

horse* OR equine OR cow OR cows OR bovine OR goat OR goats OR sheep OR ovine OR canine OR dog OR dogs OR feline OR cat OR cats)) **OR** (ts=("hydroxymethylglutaryl coenzyme A reductase inhibitor" OR "Hydroxymethylglutaryl-CoA Reductase Inhibitors" OR "Hydroxymethylglutaryl-CoA Reductase Inhibitor" OR "HMG-CoA Reductase Inhibitors" OR "HMG-CoA Reductase Inhibitor" OR "HMG CoA Reductase Inhibitors" OR "HMG CoA Reductase Inhibitor" OR "Statins" OR "Statin" OR "Hydroxymethylglutaryl-CoA Inhibitors" OR "Hydroxymethylglutaryl-CoA Inhibitor" OR "Hydroxymethylglutaryl-Coenzyme A Inhibitors" OR "Hydroxymethylglutaryl-Coenzyme A Inhibitor" OR "2,6,7-trideoxy-7-C-(2,4-dichlorophenyl)heptonic acid" OR "2-(2-(3-hydroxy-12-(2-methyl-1-oxobutoxy)-5-androstan-17-yl)ethyl)tetrahydro-4-hydroxy-2H-pyran-6-one" OR "3,24-dihydroxycholest-8(14)-en-15-one" OR "3,25-dihydroxycholest-8(14)-en-15-one" OR "3,4-dihydroxyphenylpropionylleucinamide" OR "3-hydroxy-24-dimethylaminochol-8(14)-en-15-one" OR "3-hydroxy-25,26,26,26,27,27,27-heptafluoro-5-cholest-8(14)-en-15-one" OR "3-hydroxycholest-8(14),24-dien-15-one" OR "6-(2,4-dichlorophenyl)-2,3,4-trihydroxyhexanesulfonic acid" OR "6-(2,4-dichlorophenyl)-2,4-dihydroxyhexane-1-sulfonic acid" OR "6-(2,4-dichlorophenyl)-erythro-2,4-dihydroxyhexylphosphonic acid" OR "6-(2-(2,4-dichloro-6-hydroxyphenyl)ethyl)-3,4,5,6-tetrahydro-4-hydroxy-2H-pyran-2-one" OR "6-(2-(8-((4-fluorobenzyl)oxy)-1,2,6,7,8,8a-hexahydro-2,6-dimethyl-1-naphthyl)ethyl)-4-hydroxy-3,4,5,6-tetrahydro-2H-pyran-2-one" OR "6-hydroxyisocompactin" OR "7-(2'-((2"-methyl-1"-oxobutoxy)methyl)-1'-cyclohexyl)-3-hydroxy-5-oxoheptanoic acid" OR "7-(2,4-dichlorophenyl)-3-hydroxy-5-heptanolide" OR "7-(3-bromo-4,5-bis(4-fluorophenyl)-2-(1-methylethyl)-1H-pyrrol-1-yl)-3,5-dihydroxy-6-heptenoic acid methyl ester" OR "7-(3-hydroxy-2-methyl-1,2,3,5,6,7,8,8a-octahydro-1-naphtalenyl)-3,5-dihydroxyheptanoic acid" OR "7-(7-((1,1'-biphenyl-4-yl)methyl)-2-(4-fluorophenyl)-4,5,6,7-tetrahydro-2H-indazol-3-yl)-3,5-dihydroxy-6-heptenoic acid" OR "amlodipine, atorvastatin drug combination" OR "Atorvastatin" OR "BMY 21950" OR "cerivastatin" OR "cholest-8-ene-3,15-diol" OR "chrysosporin" OR "crilvastatin" OR "dihydrocompactin" OR "dihydromevinolin" OR "fermodulin" OR "fluvastatin" OR "GR 92549" OR "GR 95030X" OR "HR 780" OR "L 157012" OR "L 645164" OR "L 647318" OR "L 669262" OR "lanost-8-en-3-ol-24-one" OR "Lovastatin" OR "Meglutol" OR "methoxypolyethoxylated cholesterol" OR "mevastatin" OR "pannorin" OR "PD 123244-15" OR "phosphoadenosine diphosphoribose" OR "pitavastatin" OR "Pravastatin" OR "red yeast rice" OR "Rosuvastatin Calcium" OR "RP 61969" OR "Simvastatin" OR "SQ 33600" OR "SRI 62320") AND ti=("Kidney Dysfunction" OR "renal dysfunction" OR "renal dysfunction*" OR "kidney dysfunction" OR "kidney dysfunction*" OR "kidney function loss" OR "renal function loss" OR "kidney function" OR "kidney function*" OR "renal function" OR "renal function*" OR "Kidney Failure" OR "Chronic Kidney Disease" OR "Chronic Renal Disease" OR "Kidney Failure" OR "Renal Failure" OR "Proteinuria" OR "Proteinuria" OR "Albuminuria" OR "Albuminuria" OR "macroalbuminuria" OR "macro-albuminuria" OR "microalbuminuria" OR "micro-albuminuria" OR "urinary protein" OR "urinary proteins" OR "urinary albumin" OR "Glomerulus Filtration Rate" OR "Glomerular Filtration Rate" OR "eGFR" OR "e-GFR" OR "GFR" OR "Kidney Function Test") AND ts=("Clinical Trial" OR "trial" OR "rct" OR "randomized" OR "randomised") NOT ti=(("meta-analysis" OR "systematic review") NOT ("Trial" OR "rct" OR "randomized" OR "randomised")) NOT ti=(veterinary OR rabbit OR rabbits OR animal OR animals OR mouse OR mice OR rodent OR rodents OR rat OR rats OR pig OR pigs OR porcine OR horse* OR equine OR cow OR cows OR bovine OR goat OR goats OR sheep OR ovine OR canine OR dog OR dogs OR feline OR cat OR cats))

Cochrane

(("hydroxymethylglutaryl coenzyme A reductase inhibitor" OR "Hydroxymethylglutaryl-CoA Reductase Inhibitors" OR "Hydroxymethylglutaryl-CoA Reductase Inhibitor" OR "HMG-CoA Reductase Inhibitors" OR "HMG-CoA Reductase Inhibitor" OR "HMG CoA Reductase Inhibitors" OR "HMG CoA Reductase Inhibitor" OR "Statins" OR "Statin" OR "Hydroxymethylglutaryl-CoA Inhibitors" OR "Hydroxymethylglutaryl-CoA Inhibitor" OR "Hydroxymethylglutaryl-Coenzyme A Inhibitors" OR "Hydroxymethylglutaryl-Coenzyme A Inhibitor" OR "2,6,7-trideoxy-7-C-(2,4-dichlorophenyl)heptonic acid" OR "2-(2-(3-hydroxy-12-(2-methyl-1-oxobutoxy)-5-androstan-17-yl)ethyl)tetrahydro-4-hydroxy-2H-pyran-6-one" OR "3,24-dihydroxycholest-8(14)-en-15-one" OR "3,25-dihydroxycholest-8(14)-en-15-one" OR "3,4-dihydroxyphenylpropionylleucinamide" OR "3-hydroxy-24-dimethylaminochol-8(14)-en-15-one" OR "3-hydroxy-25,26,26,26,27,27,27-heptafluoro-5-cholest-8(14)-en-15-one" OR "3-hydroxycholest-8(14),24-dien-15-one" OR "6-(2,4-dichlorophenyl)-2,3,4-trihydroxyhexanesulfonic acid" OR "6-(2,4-dichlorophenyl)-2,4-dihydroxyhexane-1-sulfonic acid" OR "6-(2,4-dichlorophenyl)-erythro-2,4-dihydroxyhexylphosphonic acid" OR "6-(2-(2,4-dichloro-6-hydroxyphenyl)ethyl)-3,4,5,6-tetrahydro-4-hydroxy-2H-pyran-2-one" OR "6-(2-(8-((4-fluorobenzyl)oxy)-1,2,6,7,8,8a-hexahydro-2,6-dimethyl-1-naphthyl)ethyl)-4-hydroxy-3,4,5,6-tetrahydro-2H-pyran-2-one" OR "6-hydroxyisocompactin" OR "7-(2'-((2"-methyl-1"-oxobutoxy)methyl)-1'-cyclohexyl)-3-hydroxy-5-oxoheptanoic acid" OR "7-(2,4-dichlorophenyl)-3-hydroxy-5-heptanolide" OR "7-(3-bromo-4,5-bis(4-fluorophenyl)-2-(1-methylethyl)-1H-pyrrol-1-yl)-3,5-dihydroxy-6-heptenoic acid methyl ester" OR "7-(3-hydroxy-2-methyl-1,2,3,5,6,7,8,8a-octahydro-1-naphtalenyl)-3,5-dihydroxyheptanoic acid" OR "7-(7-((1,1'-biphenyl-4-yl)methyl)-2-(4-fluorophenyl)-4,5,6,7-tetrahydro-2H-indazol-3-yl)-3,5-dihydroxy-6-heptenoic acid" OR "amlodipine, atorvastatin drug combination" OR "Atorvastatin" OR "BMY 21950" OR "cerivastatin" OR "cholest-8-ene-3,15-diol" OR "chrysosporin" OR "crilvastatin" OR "dihydrocompactin" OR "dihydromevinolin" OR "fermodulin" OR "fluvastatin" OR "GR 92549" OR "GR 95030X" OR "HR 780" OR "L 157012" OR "L 645164" OR "L 647318" OR "L 669262" OR "lanost-8-en-3-ol-24-one" OR "Lovastatin" OR "Meglutol" OR "methoxypolyethoxylated cholesterol" OR "mevastatin" OR "pannorin" OR "PD 123244-15" OR "phosphoadenosine diphosphoribose" OR "pitavastatin" OR "Pravastatin" OR "red yeast rice" OR "Rosuvastatin Calcium" OR "RP 61969" OR "Simvastatin" OR "SQ 33600" OR "SRI 62320"):ti AND ("Kidney Dysfunction" OR "renal dysfunction" OR "renal dysfunction*" OR "kidney dysfunction" OR "kidney dysfunction*" OR "kidney function loss" OR "renal function loss" OR "kidney function" OR kidney function* OR "renal function" OR renal function* OR "Kidney Failure" OR "Chronic Kidney Disease" OR "Chronic Renal Disease" OR "Kidney Failure" OR "Renal Failure" OR "Proteinuria" OR "Proteinuria" OR "Albuminuria" OR "Albuminuria" OR "macroalbuminuria" OR "macro-albuminuria" OR "microalbuminuria" OR "micro-albuminuria" OR "urinary protein" OR "urinary proteins" OR "urinary albumin" OR "Glomerulus Filtration Rate" OR "Glomerular Filtration Rate" OR "eGFR" OR "e-GFR" OR "GFR" OR "Kidney Function Test"):ti,ab,kw) **OR** (("hydroxymethylglutaryl coenzyme A reductase inhibitor" OR "Hydroxymethylglutaryl-CoA Reductase Inhibitors" OR "Hydroxymethylglutaryl-CoA Reductase Inhibitor" OR "HMG-CoA Reductase Inhibitors" OR "HMG-CoA Reductase Inhibitor" OR "HMG CoA Reductase Inhibitors" OR "HMG CoA Reductase Inhibitor" OR "Statins" OR "Statin" OR "Hydroxymethylglutaryl-CoA Inhibitors" OR "Hydroxymethylglutaryl-CoA Inhibitor" OR "Hydroxymethylglutaryl-Coenzyme A Inhibitors" OR "Hydroxymethylglutaryl-Coenzyme A Inhibitor" OR "2,6,7-trideoxy-7-C-(2,4-dichlorophenyl)heptonic acid" OR "2-(2-(3-hydroxy-12-(2-methyl-1-oxobutoxy)-5-androstan-17-yl)ethyl)tetrahydro-4-hydroxy-2H-pyran-6-one" OR "3,24-dihydroxycholest-8(14)-en-15-one" OR "3,25-dihydroxycholest-8(14)-en-15-one" OR "3,4-dihydroxyphenylpropionylleucinamide" OR "3-hydroxy-24-dimethylaminochol-8(14)-en-15-one" OR "3-hydroxy-25,26,26,26,27,27,27-heptafluoro-

5-cholest-8(14)-en-15-one" OR "3-hydroxycholest-8(14),24-dien-15-one" OR "6-(2,4-dichlorophenyl)-2,3,4-trihydroxyhexanesulfonic acid" OR "6-(2,4-dichlorophenyl)-2,4-dihydroxyhexane-1-sulfonic acid" OR "6-(2,4-dichlorophenyl)-erythro-2,4-dihydroxyhexylphosphonic acid" OR "6-(2-(2,4-dichloro-6-hydroxyphenyl)ethyl)-3,4,5,6-tetrahydro-4-hydroxy-2H-pyran-2-one" OR "6-(2-(8-((4-fluorobenzyl)oxy)-1,2,6,7,8,8a-hexahydro-2,6-dimethyl-1-naphthyl)ethyl)-4-hydroxy-3,4,5,6-tetrahydro-2H-pyran-2-one" OR "6-hydroxyisocompactin" OR "7-(2'-((2"-methyl-1"-oxobutoxy)methyl)-1'-cyclohexyl)-3-hydroxy-5-oxoheptanoic acid" OR "7-(2,4-dichlorophenyl)-3-hydroxy-5-heptanolide" OR "7-(3-bromo-4,5-bis(4-fluorophenyl)-2-(1-methylethyl)-1H-pyrrol-1-yl)-3,5-dihydroxy-6-heptenoic acid methyl ester" OR "7-(3-hydroxy-2-methyl-1,2,3,5,6,7,8,8a-octahydro-1-naphtalenyl)-3,5-dihydroxyheptanoic acid" OR "7-(7-((1,1'-biphenyl-4-yl)methyl)-2-(4-fluorophenyl)-4,5,6,7-tetrahydro-2H-indazol-3-yl)-3,5-dihydroxy-6-heptenoic acid" OR "amlodipine, atorvastatin drug combination" OR "Atorvastatin" OR "BMY 21950" OR "cerivastatin" OR "cholest-8-ene-3,15-diol" OR "chrysosporin" OR "crilvastatin" OR "dihydrocompactin" OR "dihydromevinolin" OR "fermodulin" OR "fluvastatin" OR "GR 92549" OR "GR 95030X" OR "HR 780" OR "L 157012" OR "L 645164" OR "L 647318" OR "L 669262" OR "lanost-8-en-3-ol-24-one" OR "Lovastatin" OR "Meglutol" OR "methoxypolyethoxylated cholesterol" OR "mevastatin" OR "pannorin" OR "PD 123244-15" OR "phosphoadenosine diphosphoribose" OR "pitavastatin" OR "Pravastatin" OR "red yeast rice" OR "Rosuvastatin Calcium" OR "RP 61969" OR "Simvastatin" OR "SQ 33600" OR "SRI 62320"):ti,ab,kw AND ("Kidney Dysfunction" OR "renal dysfunction" OR "renal dysfunction*" OR "kidney dysfunction" OR "kidney dysfunction*" OR "kidney function loss" OR "renal function loss" OR "kidney function" OR kidney function* OR "renal function" OR renal function* OR "Kidney Failure" OR "Chronic Kidney Disease" OR "Chronic Renal Disease" OR "Kidney Failure" OR "Renal Failure" OR "Proteinuria" OR "Proteinuria" OR "Albuminuria" OR "Albuminuria" OR "macroalbuminuria" OR "macro-albuminuria" OR "microalbuminuria" OR "micro-albuminuria" OR "urinary protein" OR "urinary proteins" OR "urinary albumin" OR "Glomerulus Filtration Rate" OR "Glomerular Filtration Rate" OR "eGFR" OR "e-GFR" OR "GFR" OR "Kidney Function Test"):ti)
